# Enhanced HIF-1α cooperation by a human RORγt mutant potentiates Th17 pathogenicity

**DOI:** 10.1016/j.celrep.2026.117123

**Published:** 2026-03-17

**Authors:** Peixian Dong, Liang Liu, John Yu, Ning Li, Benjamin Davidorf, Steven Shen, Ronald Bouch, Jing Zhang, David Ho, Gutian Xiao, Omid Akbari, Bing Zhang, Zhiheng He

**Affiliations:** 1 Department of Immunology and Immune Therapeutics, Keck School of Medicine, University of Southern California, Los Angeles, CA, USA; 2 Norris Comprehensive Cancer Center, Keck School of Medicine, University of Southern California, Los Angeles, CA, USA; 3 Department of Immunology & Theranostics, Arthur Riggs Diabetes & Metabolism Research Institute, Beckman Research Institute of the City of Hope, Duarte, CA, USA; 4 Department of Molecular Microbiology and Immunology, Wake Forest School of Medicine, Wake Forest University, Winston-Salem, NC, USA; 5 Department of Medicine, Division of Gastrointestinal and Liver Diseases, Keck School of Medicine, University of Southern California, Los Angeles, CA, USA; 6 These authors contributed equally; 7 Lead contact

## Abstract

T helper 17 (Th17) cells are pivotal in mucosal defense and autoimmune pathology, with their function governed by the transcription factor retinoic acid receptor-related orphan receptor gamma t (ROR**γ**t). Although genome-wide association studies link *RORC* variants to inflammatory diseases, their functional consequences remain poorly understood. We identify a pathogenic ROR**γ**t mutation N277D (mouse homolog N275D) that amplifies Th17 pathogenicity through cooperation with hypoxia-inducible factor HIF-1**α**. This mutation enhances IFN-**γ** and other Th1-type cytokine production by Th17 cells, exacerbating colitis without disrupting T cell development or homeostasis. Integrated transcriptomic and metabolomic profiling reveals activation of glycolytic and hypoxia-associated pathways, consistent with increased ROR**γ**t^N275D^ recruitment by HIF-1**α** to the *Pdk1* locus. Notably, silencing *Pdk1* normalizes the excessive IFN-**γ** production in ROR**γ**t^N275D^ Th17 cells. Together, these findings define a regulatory axis linking ROR**γ**t and HIF-1**α** that coordinates transcriptional and metabolic programs in pathogenic Th17 cells, providing a framework for dissecting the functional impact of autoimmune risk variants.

## In brief

Dong et al. report a single ROR**γ**t mutation that rewires Th17 metabolism and cytokine output, fueling colitis through a HIF-1**α**-PDK1 axis. Blocking PDK1 restores immune balance, pointing to a new therapeutic target in pathogenic Th17 cells.

## Graphical Abstract

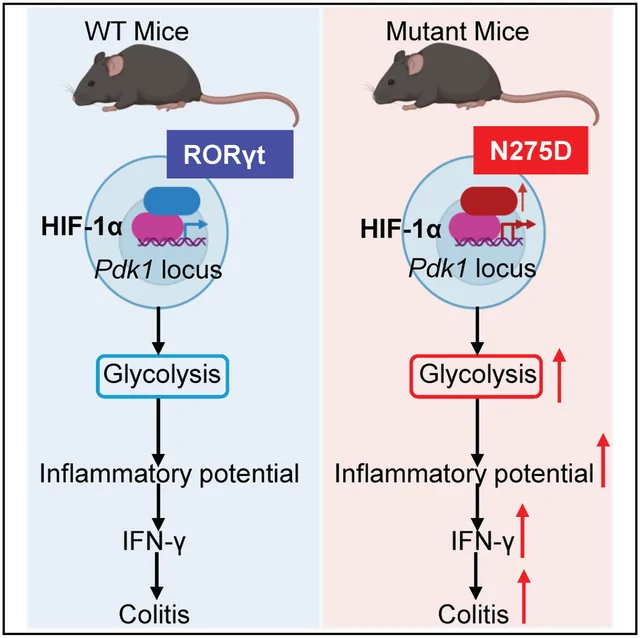

## INTRODUCTION

IL-17-producing T helper 17 (Th17) cells are essential mediators of mucosal immunity, maintaining epithelial barrier integrity and protecting the host against extracellular bacteria and fungi.^1,2^ Beyond their canonical functions, Th17 cells can acquire a proinflammatory program characterized by the expression of Th1-type cytokines such as IFN-γ, which amplifies their pathogenic potential and contributes to the development of chronic inflammatory diseases, including inflammatory bowel disease (IBD), multiple sclerosis, and psoriasis.^1,3,4^ This functional reprogramming underscores the remarkable plasticity of Th17 cells and highlights the importance of transcriptional and metabolic networks in determining Th17 responses.^5,6^

The transcription factor retinoic acid receptor-related orphan receptor gamma t (RORγt) is the lineage-defining regulator of Th17 cells.^7,8^ RORγt is indispensable for Th17 differentiation, directly controlling the expression of cytokines such as IL-17A, IL-17F, and IL-22.^7,9^ RORγt serves as a central integration node for extracellular signals and intracellular pathways that fine-tune Th17 identity and effector function.^7,10–12^ Pharmacological targeting of RORγt has shown promise in preclinical autoimmune models, positioning it as a candidate for therapeutic intervention.^13–15^ However, the molecular mechanisms by which RORγt balances homeostatic and inflammatory programs remain incompletely understood.

RORγt contains three functional domains: a DNA-binding domain, a hinge region, and a ligand-binding domain (LBD).^16^ Together, these domains regulate DNA interaction, nuclear localization, and cofactor recruitment. Post-translational modifications (PTMs) further modulate RORγt’s transcriptional activities, with ubiquitylation and phosphorylation known to affect the balance between homeostatic and inflammatory Th17 responses.^17–20^ The relevance of RORγt to human disease is underscored by loss-of-function mutations in *RORC* that cause primary immunodeficiency with recurrent fungal infections due to defective Th17 responses,^21^ as well as by genome-wide association studies (GWASs) identifying *RORC* polymorphisms associated with autoimmune disorders.^22^ Despite these associations, the functional impact of most disease-linked variants has not been established.

Emerging evidence indicates that RORγt cooperates with other transcriptional regulators to shape Th17 function, with hypoxia-inducible factor 1α (HIF-1α) as a central partner. HIF-1α drives glycolysis, a pathway essential for sustaining the proinflammatory activity of Th17 cells,^23–26^ and its interaction with RORγt co-occupies lineage-defining loci to amplify the expression of cytokines such as IL-17 and IL-22.^25,26^ This partnership integrates environmental cues, including hypoxia and nutrient availability, with transcriptional programs that fuel inflammatory potential.^27–29^ Together, HIF-1α and RORγt establish a feedforward circuit in which HIF-1α promotes glycolytic enzyme expression while RORγt reinforces cytokine production, coupling metabolism to effector function.^27,28^ Yet, it remains unclear how mutations in RORγt alter this interaction or whether changes in RORγt-HIF-1α binding selectively reprogram metabolic pathways that sustain autoimmune inflammation.

To address this gap, we performed an unbiased screen of RORγt variants reported in public databases and GWAS datasets. We identified a single amino acid substitution in the LBD, asparagine to aspartate (N277D in human and N275D in mouse), that unexpectedly conferred a pathogenic phenotype. This variant enhanced Th17 cytokine production, promoted inflammatory reprogramming, and exacerbated colitis in a gene dosage-dependent manner. Mechanistically, the RORγt-N275D mutant displayed increased affinity for HIF-1α, leading to augmented transcription of glycolytic enzymes, including PDK1, and reinforcing a metabolic state that fuels proinflammatory cytokine production. Together, this work highlights how disease-associated RORγt mutations can fuel Th17 inflammatory potential and underscores the need for mutation-specific therapeutic strategies.

## RESULTS

### Identification and functional characterization of hyperactive human RORγt variants

To explore how natural genetic variation might shape RORγt activity and Th17-driven inflammation, we conducted a focused screening of human RORC (RORγt) variants. RORγt is indispensable for thymocyte survival and Th17 differentiation, and mutations that enhance its activity may underlie heightened immune responses in inflammatory disease.

**Figure 1.**
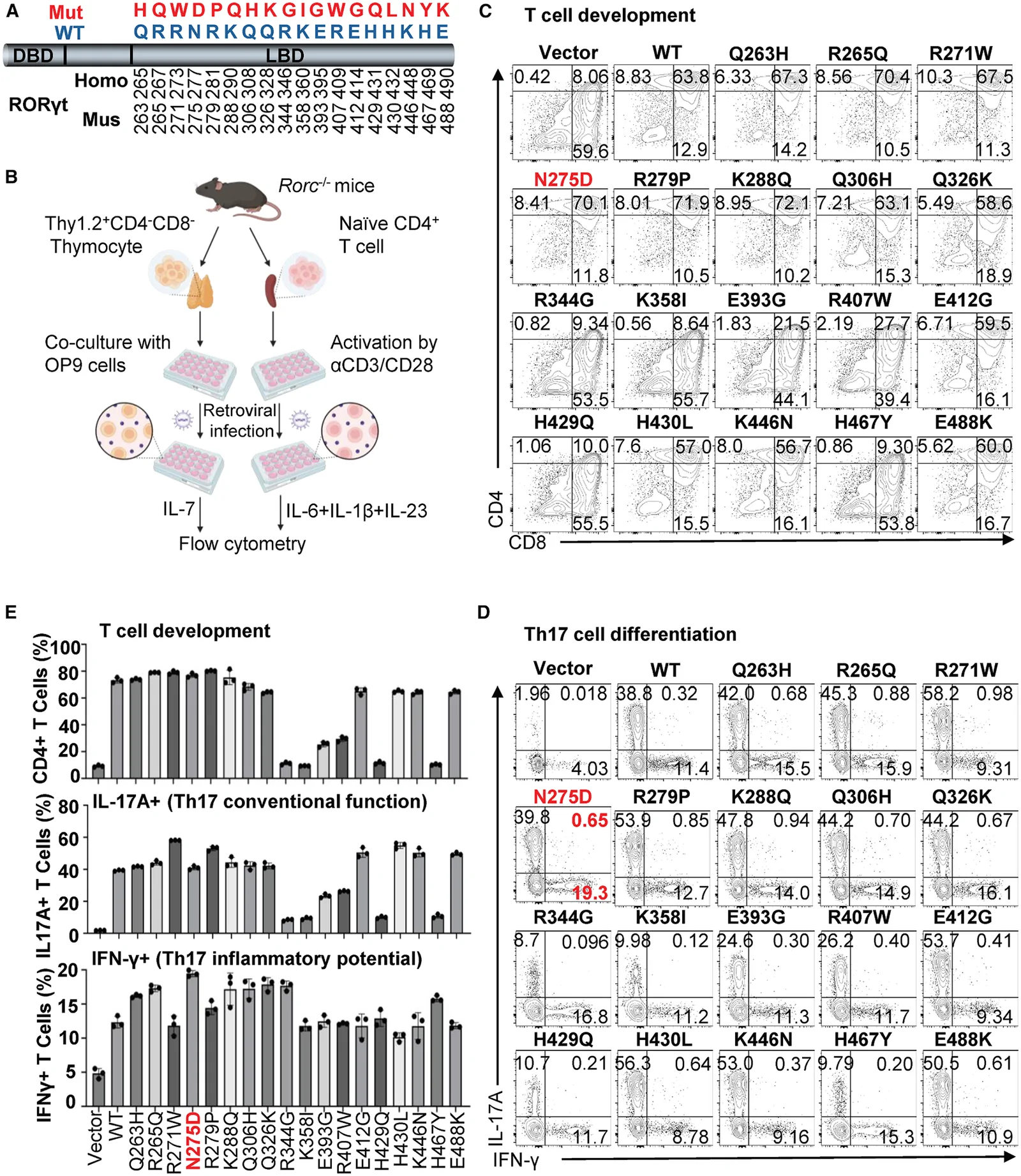
Identification and functional characterization of hyperactive human RORγt variants (A) Schematic representation of RORγt domain structure and the locations of the selected LBD variants. Numbers indicate amino acid positions. DBD, DNA-binding domain and LBD, ligand-binding domain. (B) Experimental design of *in vitro* development and differentiation assays. *Rorc*^−/−^ thymic Thy1.2^+^CD4^−^CD8^−^ double-negative (DN) progenitors or naive CD4^+^ T cells were co-cultured with OP9-DL4 stromal monolayers or stimulated with αCD3/CD28, followed by retroviral transduction with WT or mutant RORγt and subsequent culture with IL-7 (*in vitro* development) or under polarization conditions (IL-6+IL-1β+IL-23; *in vitro* differentiation). Cell surface marker expression and cytokine production were analyzed by flow cytometry. (C) Representative flow plots of *in vitro* thymocyte development from *Rorc*^−/−^ CD4^−^CD8^−^ DN progenitors retrovirally expressing WT or the indicated RORγt mutant. (D) Representative flow plots of IL-17A and IFN-γ in murine *Rorc*^−/−^ naive CD4^+^ T cells transduced with retroviruses encoding WT or the indicated RORγt mutants and cultured under pathogenic Th17-polarizing conditions (IL-6, IL-1β, and IL-23). (E) Quantification of the results shown in (C) and (D). Top, percentage of CD4^+^ thymocytes; middle, percentage of IL-17A^+^ Th17 cells; and bottom, percentage of IFN-γ^+^ Th17 cells across variants relative to WT (defined as 100%). All data reported as mean ± SD, *n* = 3 biological replicates per group. See also Figure S1.

We compiled all known RORC coding variants from population databases (IGSR, The International Genome Sample Resource. NCBI identifiers: human RORC gene: 6097 and mouse Rorc gene: 19885) and applied a stepwise computational filter that prioritized (1) missense substitutions; (2) variants within the LBD, which regulates transcriptional activity and cofactor interaction; and (3) residues with high conservation and predicted functional impact. Because the LBD is the target of several allosteric modulators, alterations in this domain are especially likely to influence function. This analysis yielded 18 top candidates (Figure 1A), which were evolutionarily conserved between human and mouse (Figure S1A). Each variant was introduced into retroviral constructs and expressed in primary T cells from *Rorc*^−/−^ mice. Protein levels were comparable across variants (Figure S1B), allowing downstream functional comparisons.

We first assessed thymocyte development, a context in which RORγt promotes survival of CD4^+^CD8^+^ double-positive (DP) cells. *Rorc*-deficient hematopoietic progenitor cells (CD4^−^CD8^−^ double-negative [DN]) were transduced with each variant and developed toward DP and eventually CD4 or CD8 single-positive (SP) T cells *in vitro*^20^ (Figures 1B and 1C). Several variants, including N275D, restored DP and SP T cell development, comparable to wild-type (WT) RORγt, whereas others (R344G, K358I, H429Q, and H467Y) were unable to promote T cell maturation (Figures 1C, 1E, and S1C).

We next assessed peripheral Th17 differentiation. Naive *Rorc*^−/−^ CD4^+^ T cells were transduced with each variant and polarized using IL-6, IL-1β, and IL-23^20,30,31^ (Figures 1B and 1D). This cytokine combination is known to promote the differentiation of highly inflammatory Th17 cells implicated in autoimmunity and tissue inflammation, which express both IL-17 and Th1 cytokines, including IFN-γ. Although variants defective in thymocyte assays also failed to restore IL-17A expression under Th17-polarizing conditions (Figures 1D, 1E, and S1D), several variants supported IL-17A production at levels comparable to WT. Notably, a subset of these functional variants aberrantly increased IFN-γ production, a hallmark of pathogenic Th17 cells. Among them, N275D elicited the strongest IFN-γ response (Figure 1E), defining it as a pathogenic allele with potential clinical significance. While additional variants may also warrant future investigation, our findings highlight naturally occurring RORγt variants that enhance Th17 inflammatory potential, with N275D standing out as the most potent.

### RORγt^N275D^ KI mice exhibit normal development and steady-state immunity

**Figure 2.**
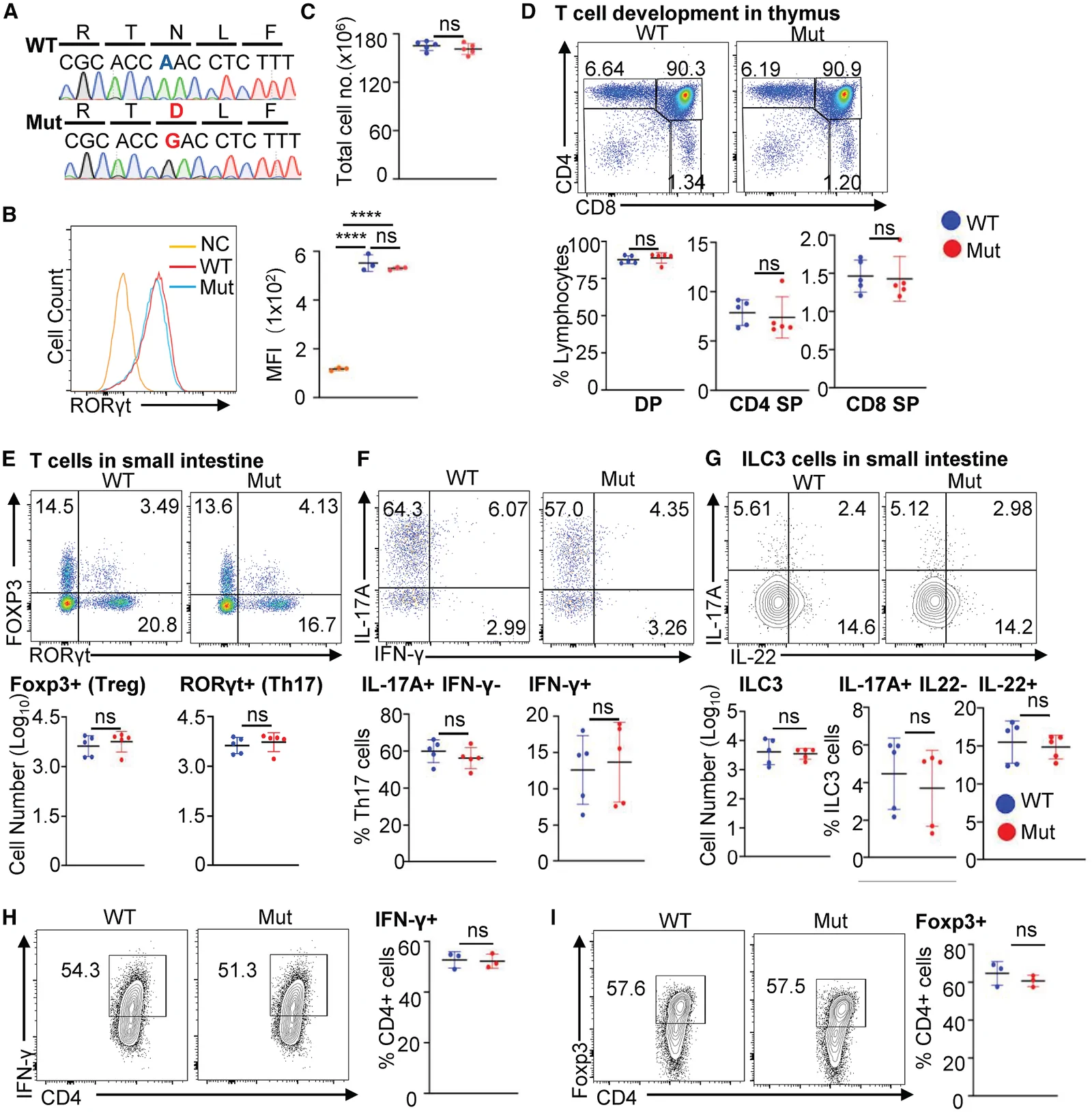
RORγt^N275D^ knockin mice exhibit normal T cell development and immune homeostasis at steady state (A) Sanger chromatograms confirm the codon change in the KI allele. (B) Flow cytometric analysis of RORγt expression in *in vitro* differentiated Th17 cells from WT and mutant mice (*n* = 3 biological replicates per group). (C) Quantification of RORγt expression and total thymic cellularity (*n* = 5 biological replicates per group). (D) Flow cytometry analysis (left) of thymocyte development in WT and mutant mice; Statistics of frequencies (right) of DP, CD4 SP, and CD8 SP populations (*n* = 5 biological replicates per group). (E) Small-intestinal lamina propria CD4^+^ T cells intracellular staining with RORγt and Foxp3 (left); statistics of total numbers (right) of Foxp3^+^ (Treg) and RORγt^+^ (Th17) cells (*n* = 5 biological replicates per group). (F) Flow cytometry analysis (left) of E RORγt^+^ (Th17); statistics of frequencies (right) of IL-17A SP and IFN-γ^+^ cells (*n* = 5 biological replicates per group). (G) Flow cytometry analysis (left) of ILC3 cells of the small intestine; statistics of frequencies (right) of IL-17A^+^ SP and IL-22^+^ cells (*n* = 5 biological replicates per group). (H) Flow cytometer analysis of IFN-γ^+^ cells in naive CD4^+^ cells differentiated under Th1 priming conditions for three days. Right: quantification of the results (*n* = 3 biological replicates per group. (I) Flow cytometer analysis of Foxp3^+^ cells in naive CD4^+^ cells differentiated under Treg priming conditions for three days. Right: quantification of the results (*n* = 3 biological replicates per group). Statistical significance was determined using unpaired Student’s *t* test. ****p < 0.0001; ns, not significant; All data reported as mean ± SD. See also Figure S2.

To evaluate the physiological relevance of this hyperactive allele, we generated RORγt^N275D^ knockin (KI) mice using CRISPR-Cas9 (Figure S2A). The residue is highly conserved (Figure S1A), supporting a murine model to assess its impact. Sanger sequencing confirmed precise editing (Figure 2A), and RORγt^N275D^ protein levels in Th17 cells were comparable to WT (Figure 2B). KI mice were born at Mendelian ratios without gross abnormalities. Thymocyte profiling showed normal cellularity and distribution of DN, DP, CD4^+^, and CD8^+^ SP subsets in KI versus WT mice (Figures 2C, 2D, and S2B–S2E), consistent with *in vitro* thymocyte assays, indicating that N275D preserves RORγt’s essential developmental role.

We next examined intestinal immunity, where RORγt is critical for tissue-resident Th17 cells. The N275D mutation did not alter the frequency or IL-17A production of CD4^+^RORγt^+^ T cells in the spleen or mesenteric lymph nodes (Figures S2F–S2G). In the lamina propria, both the frequencies and total numbers of CD4^+^RORγt^+^ cells were comparable between KI and WT mice, as were their production of IL-17A and IFN-γ (Figures 2E, 2F, and S2H).

Intestinal CD4^+^Foxp3^+^ Treg numbers were also similar (Figure 2E), indicating that RORγt^N275D^ does not perturb immune homeostasis under steady-state conditions. Because RORγt^+^ also governs the development and function of group 3 innate lymphoid cells (ILC3s), we further assessed ILC3 populations in the intestinal lamina propria. Both the frequency and number of RORγt^+^ ILC3 cells, as well as their production of IL-17A and IFN-γ, were comparable between KI and WT mice (Figures 2G and S2I). Of note, *in vitro* differentiation assays showed that the N275D mutation does not alter Th1 or Treg lineage commitment (Figures 2H and 2I).

### RORγt^N275D^ enhances Th17 inflammatory potential *in vivo*

To directly evaluate the *in vivo* impact of the pathogenic RORγt^N275D/N275D^ variant, we employed the T cell transfer colitis model, a well-established system for probing Th17-driven inflammation.^32^ In this model, naive CD45^+^CD3^+^CD4^+^CD25^−^ CD45RB^hi^ T cells are transferred into *Rag1*^−/−^ mice, which lack endogenous T and B cells, thereby allowing precise assessment of T cell-intrinsic effects independent of other RORγt-expressing populations such as ILC3s or γδ T cells.

**Figure 3.**
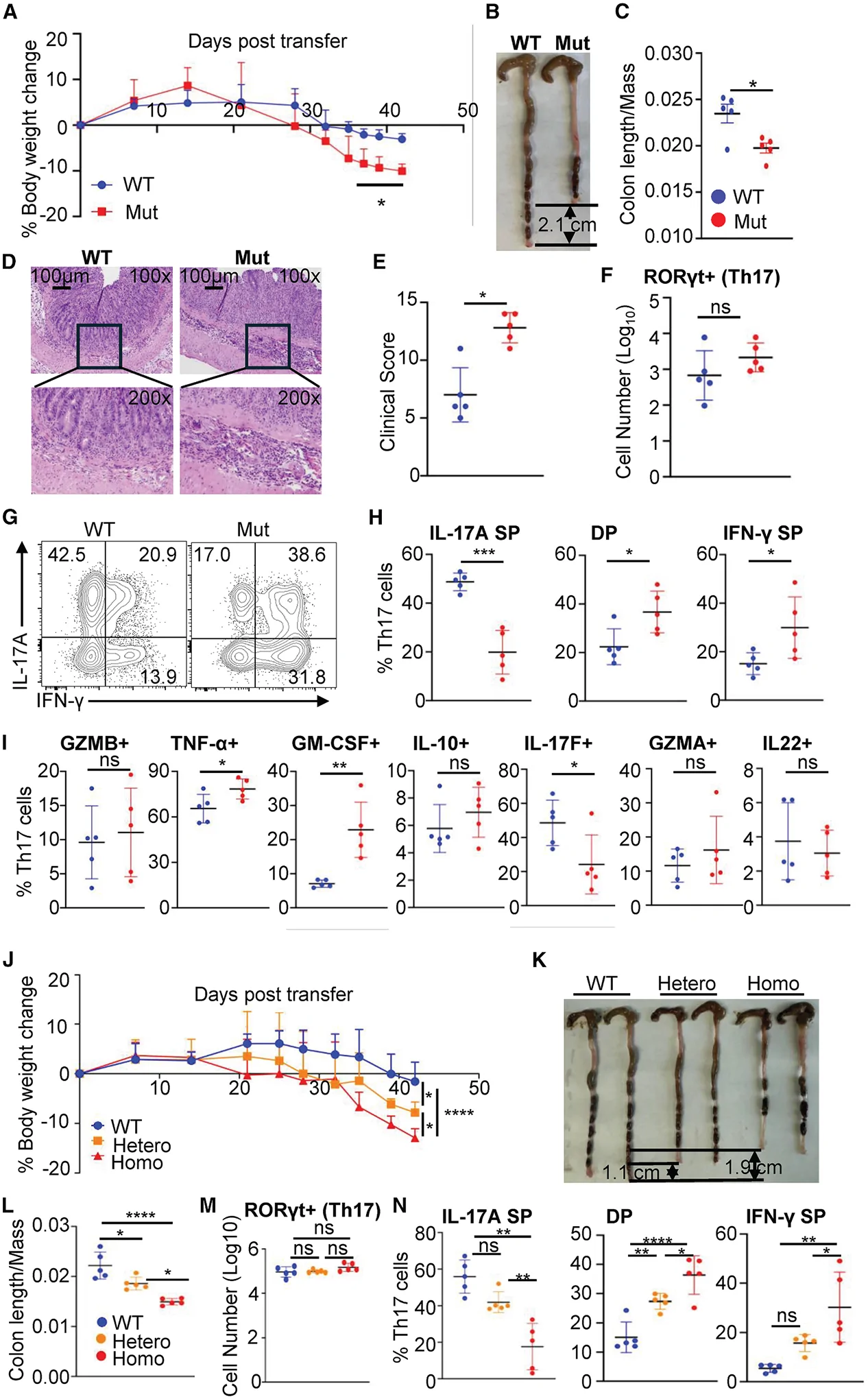
RORγt^N275D^ enhances Th17 inflammatory potential *in vivo* in a dose-dependent manner (A) *Rag1*^−/−^ recipients were transferred with naive CD4^+^ T cells isolated from WT or RORγt^N275D^ knockin donors. Body weight change of transfer colitis recipients reported as a percentage of starting body weight at day 0. (B and C) Representative image (B) and statistics of length/mass ratio (C) of the colon from transfer colitis recipients between WT or mutant donors’ group. (D and E) Representative H&E-stained colon sections from transfer colitis mice (D), with corresponding histology scores (E). (F–H) Absolute number of Th17 cells (F), representative flow cytometry plot of colonic IL-17A SP, DP, and IFN-γ SP Th17 cells (G), and statistics of frequency of IFN-γ SP, DP, or IL-17A SP Th17 (H) cells from the lamina propria from transfer colitis recipients’ mice. (I) Statistics of frequency of GZMB^+^, TNF-α^+^, GM-CSF^+^, IL-10^+^, IL-17F^+^, GZMA^+^, and IL-22^+^ Th17 (G) cells from the lamina propria from transfer colitis recipients’ mice. (J) *Rag1*^−/−^ recipients were transferred with naive CD4^+^ T cells isolated from WT, RORγt^N275D^ heterozygous, or RORγt^N275D^ homozygous. Body weight change of transfer colitis recipients reported as the percentage of starting body weight at day 0. (K) Representative images of colons from transfer colitis recipients reconstituted with WT, RORγt^N275D^ heterozygous, or RORγt ^N275D homozygous donor T cells. (L) Statistics of the length/mass ratio of the colon from transfer colitis recipients among WT, RORγt^N275D^ heterozygous, or RORγt^N275D^ homozygous donors’ group. (M and N) Absolute number of Th17 cells (M) and statistics of frequency of IFN-γ SP, DP, or IL-17A SP Th17 (J) cells from the lamina propria from transfer colitis recipients’ mice. Statistical significance was determined using unpaired, multiple Student’s *t* test, or one-way ANOVA. *p < 0.05, **p < 0.01, ***p < 0.001, and ***p < 0.0001; ns, not significant; All data reported as mean ± SD, *n* = 5 biological replicates per group. See also Figure S3.

Naive CD4^+^ T cells from WT or RORγt^N275D^ KI mice were transferred into *Rag1*^−/−^ recipients, and body weight was monitored as a measure of disease progression. Mice receiving N275D T cells exhibited significantly greater weight loss than those receiving WT T cells (Figure 3A), consistent with exacerbated colitis. By six weeks post-transfer, colons from the N275D group were markedly shorter (Figures 3B and 3C), consistent with severe intestinal inflammation and tissue damage, as confirmed by histological analysis and clinic scores (Figures 3D and 3E).

Despite comparable frequencies of lamina propria Th17 cells (CD3^+^CD4^+^CD45^+^RORγt^+^) between groups (Figures 3F and S3A), recipients of N275D T cells harbored a significantly higher proportion of IL-17A^+^IFN-γ^+^ DP cells as well as IFN-γ-SP Th17 cells (Figures 3G, 3H, and S3B). In addition to elevated IFN-γ, N275D T cells produce increased levels of multiple proinflammatory cytokines, including GM-CSF, TNFα, and IL-17F (Figures 3I; S3C). Together, these results indicate that N275D promotes a pathogenic Th17 phenotype, thereby providing a mechanistic explanation for the worsened disease *in vivo*.

### RORγt^N275D^ heterozygosity is sufficient to enhance Th17 pathogenicity

While homozygous RORγt^N275D^ mutation clearly amplifies Th17-driven inflammation, most disease-associated variants occur in heterozygotes. To address this, we evaluated the pathogenic potential of T cells from RORγt^N275D/+^ mice using the T cell transfer colitis model. Naive CD4^+^ T cells from WT, heterozygous, and homozygous mice were transferred into *Rag1*^−/−^ hosts.

Recipients of RORγt^N275D/+^ T cells exhibited greater weight loss than WT recipients, though less severe than homozygous mutants (Figure 3J), and a similar gene-dosage pattern was observed in colon length at six weeks (Figures 3K and 3L). Despite comparable numbers of lamina propria Th17 cells across groups (Figure 3M), cytokine profiling suggested a dose-dependent increase in IL-17A^+^IFN-γ^+^ DP cells as well as IFN-γ-SP Th17 cells (Figures 3N, S3D, and S3E). Together, this graded phenotypic severity across WT, heterozygous, and homozygous mice provides strong *in vivo* genetic evidence that the enhanced Th17 pathogenicity is specifically attributable to the N275D mutation itself, supporting a causal role for this allele independent of additional genetic manipulation.

### RORγt^N275D^ reprograms the Th17 transcriptional landscape

**Figure 4.**
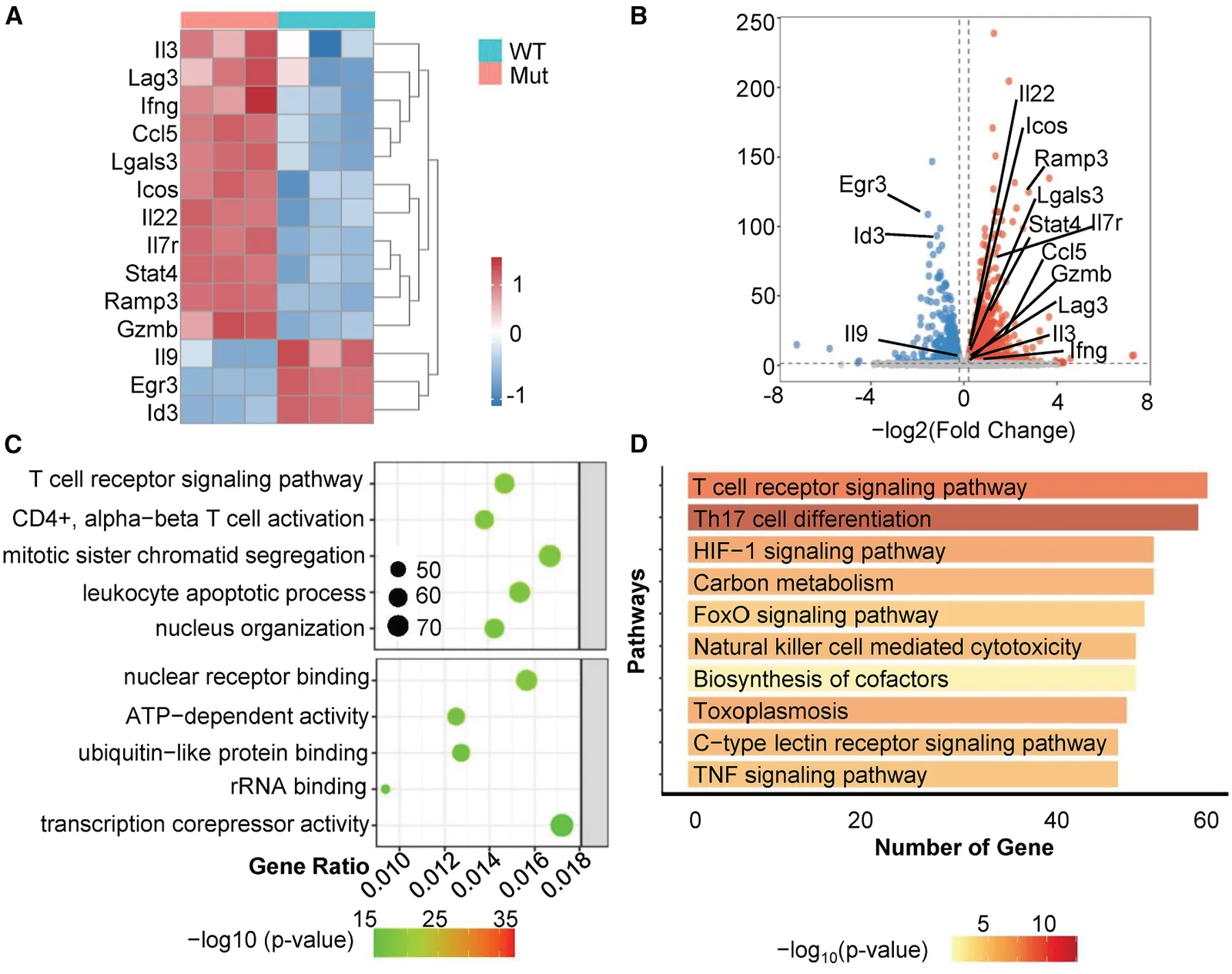
RORγt^N275D^ reprograms the transcriptional landscape of inflammatory Th17 cells (A) Heatmap visualization of pathogenic-related genes of the upregulated (red) and downregulated (blue) genes determined by RNA-seq of DEGs across WT and mutant samples. Columns are replicates; rows are genes (row-scaled *Z* scores). (B) Volcano plot of DEGs across WT and mutant group. Red, upregulated in the mutant group; blue, downregulated; and gray, not significant. (C) Gene Ontology enrichment of DEGs across the WT and mutant groups. Bubble plots show biological process (top) and molecular function (bottom); dot size indicates gene count, and color encodes significance (−log_10_*p* value). The top differentially enriched pathways were listed. (D) Kyoto Encyclopedia of Genes and Genomes pathway enrichment of DEGs across the WT and mutant groups. Bar length indicates the number of genes per pathway, and color encodes significance (−log_10_*p* value). The top differentially enriched pathways were listed. See also Figure S4.

To investigate the mechanisms driving pathogenic skewing, we profiled WT and RORγt^N275D^ Th17 cells differentiated under inflammatory conditions (IL-6, IL-1β, and IL-23)^30,31^ that mimic the environment of the colitis model. RNA sequencing (RNA-seq) revealed extensive transcriptional remodeling (Figure S4A), including strong upregulation of proinflammatory cytokine genes such as *Ifng*, *Il17a*, and *Gzmb* (Figures 4A and 4B), consistent with the enhanced pathogenicity observed *in vivo*.^33,34^ Mutant Th17 cells also showed increased expression of pathogenicity-associated genes (*Ramp3*, *Stat4*, and *Ccl5*) and reduced expression of negative regulators (*Egr3*, *Id3*, and *Il9*), reflecting a global shift toward a highly proinflammatory, tissue-aggressive state.^35–43^

Pathway analysis highlighted broad upregulation of Th17 effector programs together with striking enrichment of glycolytic pathways (Figures 4C, 4D, S4B, and S4C). Elevated glycolytic enzymes (*Gapdh*, *Hk1*, and *Pfkp*) indicated metabolic reprogramming that fuels the energy and biosynthetic demands of pathogenic Th17 cells.^44,45^ In parallel, hypoxia-responsive genes were upregulated, pointing to the activation of a hypoxia-driven transcriptional program. Because hypoxia stabilizes HIF-1α, which in turn promotes glycolysis, these two signatures converge on a feedforward circuit coupling metabolism to inflammatory function.

Cycloheximide chase experiments confirmed similar protein stability between WT and mutant RORγt (Figure S4D), excluding altered degradation as the mechanism. Together, these findings indicate that the N275D mutation activates a HIF-1α-centered transcriptional network that integrates metabolic and environmental signals to amplify Th17 pathogenicity (Figure 4D).

### RORγt^N275D^ rewires Th17 metabolism toward hypoxia-associated programs

Because RNA-seq implicated metabolic rewiring, we performed targeted metabolomics on WT and RORγt^N275D^ Th17 cells using a panel of 150 metabolites spanning glycolysis, the TCA cycle, amino acid metabolism, and one-carbon metabolism, which are essential for energy, redox balance, and biosynthesis in activated T cells. Unsupervised hierarchical clustering revealed clear separation between WT and mutant cells (Figure S5A), indicating a global metabolic shift driven by the N275D mutation.

**Figure 5.**
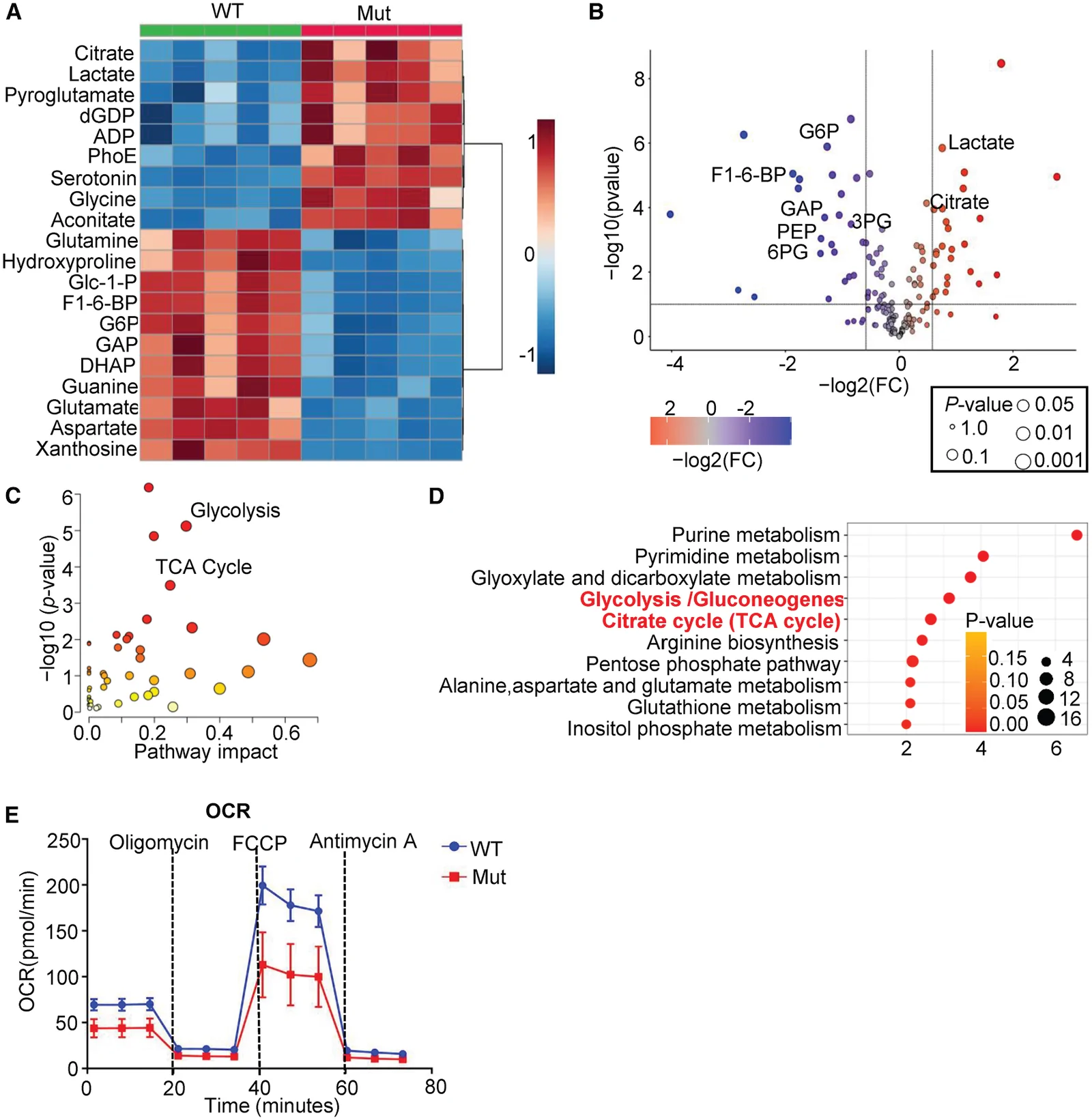
RORγt^N275D^ reshapes the metabolic profile of Th17 cells toward a hypoxia-associated program (A) Metabolomic profiling heatmap of WT and RORγt^N275D^ Th17 cells. Columns are biological replicates; rows are metabolites (row-scaled *Z* scores) clustered by similarity. The top differentially abundant metabolites were listed. (B) Volcano plot of metabolites showing the differential metabolites across the WT and mutant groups. The metabolites correlated with glycolysis and the TCA cycle are highlighted. (C) Pathway topology analysis of differentially metabolites. Bubble size denotes pathway impact; dot size indicates metabolites count, and color encodes significance (−log_10_*p* value). (D) Pathway enrichment dot plot of significantly affected metabolic pathways; dot size indicates metabolite count, and color encodes significance (−log_10_*p* value). The top differential enrichment pathways are listed. (E) Mitochondrial respiratory profile showing OCRs in response to oligomycin (ATP synthase inhibitor), BAM15 (mitochondrial uncoupler), and rotenone+antimycin A (complex I and II inhibitors) (*n* = 3 biological replicates per group). See also Figure S5.

A heatmap of the top 20 altered metabolites showed consistent upregulation of glycolytic intermediates, including glucose-6-phosphate, fructose-1,6-bisphosphate, and phosphoenolpyruvate (Figures 5A and 5B), matching the elevated glycolytic gene expression seen by RNA-seq and pointing to enhanced glycolytic flux. Pathway enrichment analysis revealed activation of glycolysis, pyruvate metabolism, and HIF-1 signaling in N275D-expressing Th17 cells (Figures 5C and 5D). Elevated levels of TCA intermediates, including citrate, succinate, and malate (Figure S5B), further suggested intensified mitochondrial activity to support biosynthetic and energetic demands. Consistent with enhanced HIF-1 signaling, these mutant Th17 cells exhibited a reduced oxygen consumption rate (OCR) (Figure 5E). Together, these results demonstrate that RORγt^N275D^ reprograms Th17 cells into a hypoxia-associated, glycolytic state that reinforces their inflammatory potential.

### RORγt^N275D^ strengthens interaction with HIF-1α to drive pathogenicity

**Figure 6.**
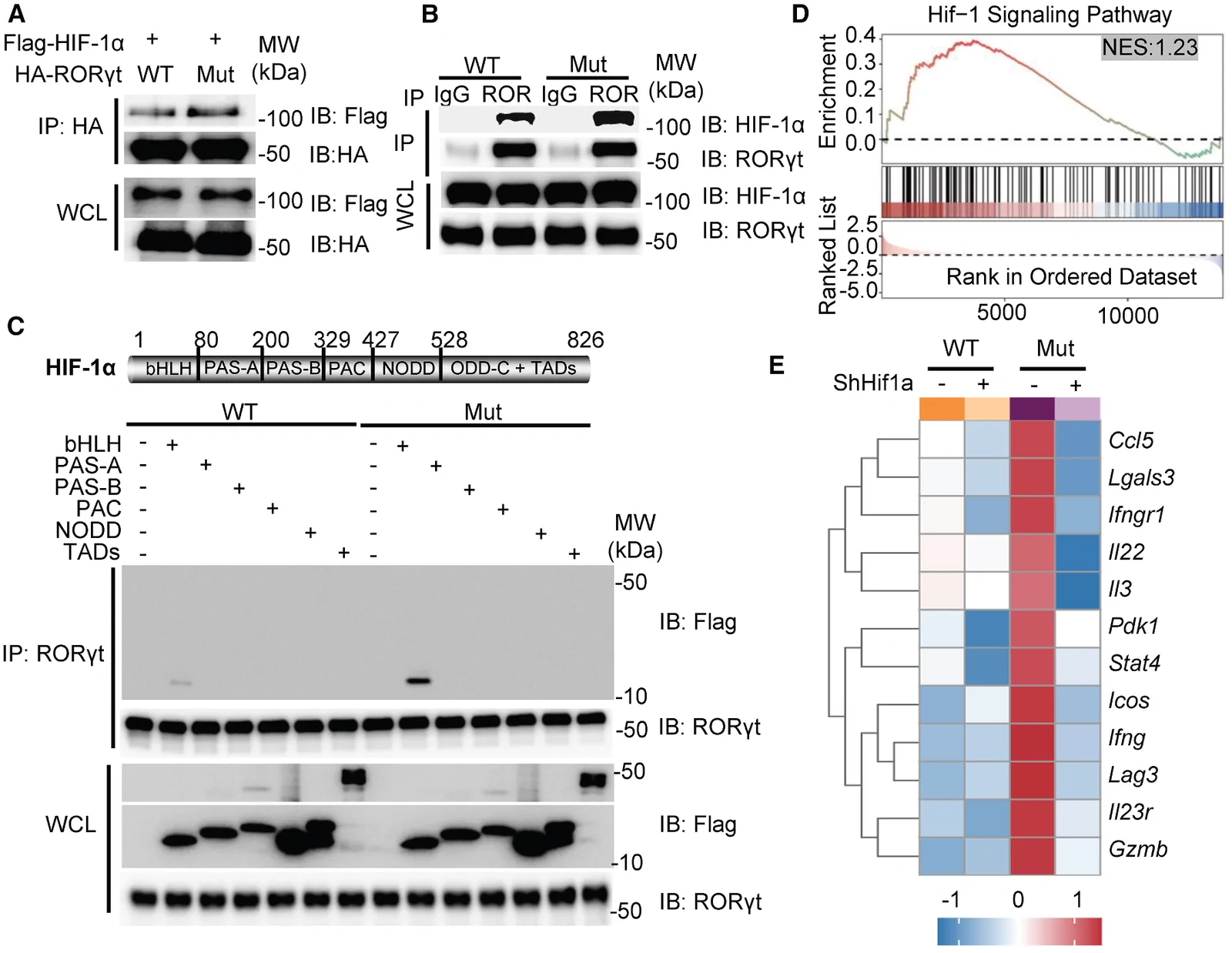
RORγtN275D strengthens interaction with HIF-1α to drive pathogenicity (A) Immunoprecipitation (IP) analysis of the interaction between WT RORγt or RORγt-N275D and HIF-1α in HEK293T cells transfected with the indicated expression plasmids. (B) IP detection of interaction between WT RORγt or RORγt-N275D and HIF-1α in the *in vitro* differentiation of Th17 cells. (C) Top, schematic of HIF-1α domain organization and amino acid boundaries. Indicated HIF-1α corresponding fragments (± denotes inclusion of each domain) were coexpressed with either WT or mutant (Mut RORγt). RORγt was immunoprecipitated (IP: RORγt), and the associated FLAG-tagged HIF-1α fragments were detected by immunoblotting (IB: FLAG). IB: RORγt shows IP efficiency. Whole-cell lysates (WCL) were immunoblotted for FLAG and RORγt as input controls. MW, molecular weight (kDa). (D) Gene set enrichment analysis of RNA-seq data showing significant enrichment of the HIF-1 signaling pathway in RORγt^N275D^ Th17 cells (NES ≈1.23). (E) Heatmap showing pathogenic gene expression rescued by *Hif1a* knockdown in pTH17 cells. WT and Mut pTH17 cells were independently transduced with *Hif1a* shRNA-expressing retrovirus (or control), and the expression of pathogenic signature genes was quantified and visualized as row-scaled *Z* scores (*n* = 3 biological replicates per group). See also Figure S6.

To connect transcriptional and metabolic changes mechanistically, we tested whether N275D alters RORγt’s interaction with HIF-1α. Co-immunoprecipitation in 293T cells and primary Th17 cells showed that RORγt^N275D^ binds more strongly to HIF-1α than WT (Figures 6A and 6B). HIF-1α contains six domains, including basic-helix-loop-helix (bHLH), PAS-A, PAS-B, PAC, NODD, and ODDC/TADs. Consistent with previous studies, the bHLH domain mediated RORγt binding. Notably, this interaction was dramatically enhanced by the N275D mutation (Figure 6C). Correspondingly, RORγt^N275D^ Th17 cells upregulated numerous HIF-1α target genes (Figure 6D). Importantly, silencing *Hif1a* in N275D-expressing Th17 cells (Figure S6B) restored pathogenic gene expression to baseline levels (Figure 6E), indicating that enhanced RORγt-HIF-1α binding promotes transcriptional activation of the HIF-1 signaling pathway and reinforces metabolic programs that fuel inflammatory Th17 responses. Intriguingly, enhanced N275D-HIF-1α interaction slightly stabilized HIF-1α, although it might not explain the increased Th17 pathogenic potential (Figure S6C).

### RORγt^N275D^-HIF-1α elevates PDK1 expression

**Figure 7.**
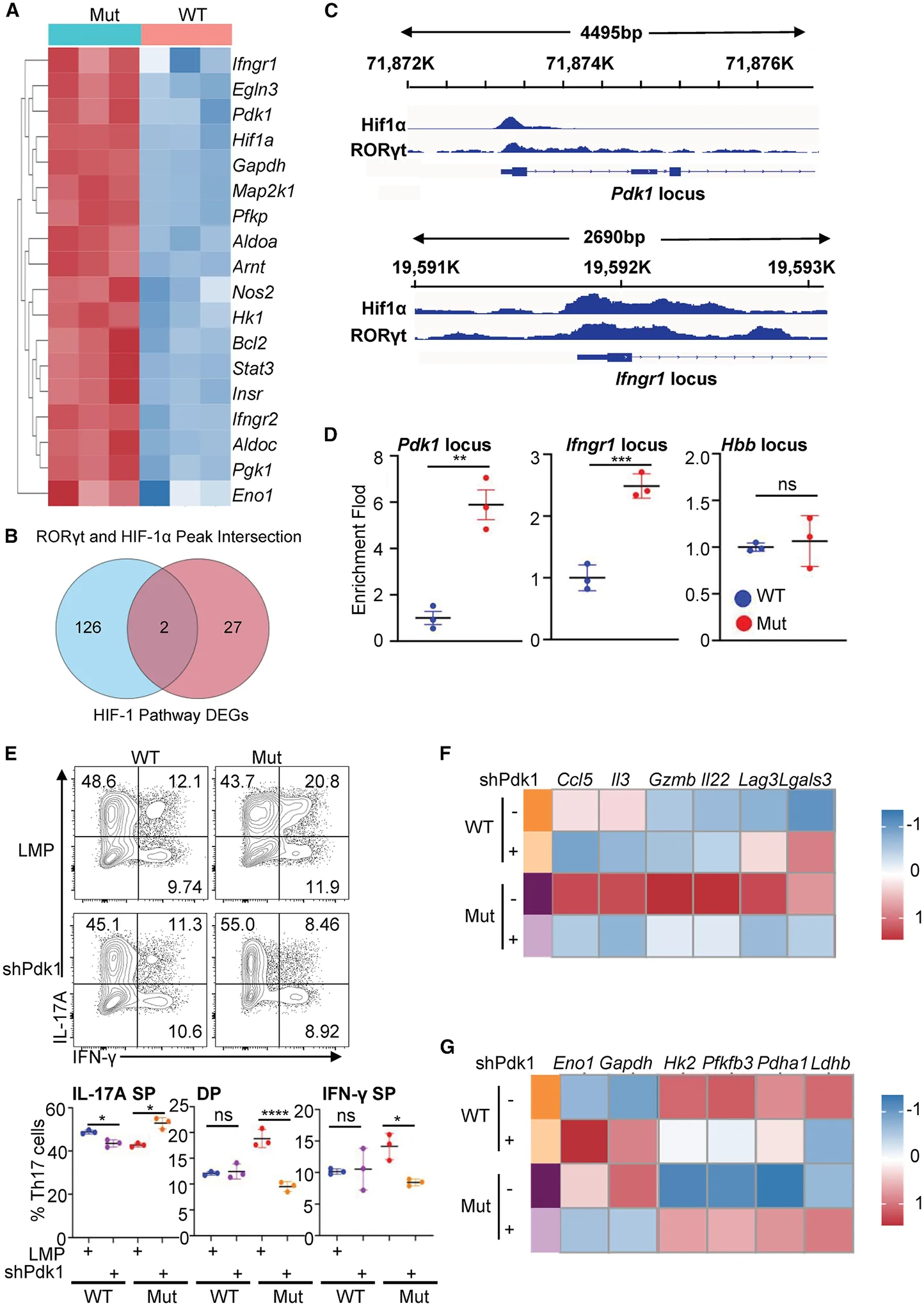
RORγt^N275D^-HIF-1α elevates PDK1 expression (A) Heatmap depicting the expression of HIF-1α signaling pathway-associated genes from RNA-seq analysis comparing WT and RORγt-N275D mutant Th17 cells. The top differential genes were listed. (B) Venn diagram showing the overlap between RORγt and HIF-1α peak intersections derived from public Cistrome ChIP-seq datasets and HIF-1 pathway DEGs identified from our RNA-seq analysis in Figure 4. (C) Cistrome database ChIP assay identified HIF-1a and RORγt DNA-binding peaks in *Pdk1* and *Ifngr1* loci. (D) ChIP-qPCR analysis showing increased recruitment of RORγt-N275D to the HIF-1α binding site at the *Pdk1* and *Ifngr1* locus, while enrichment at the negative control region (*Hbb*) was comparable between WT and N275D, indicating similar off-target/background signal. (E) Representative flow cytometry plots (top) and quantification (bottom) of IL-17ASP, DP, and IFN-γ SP Th17 cells following pathogenic Th17 polarization in cells transduced with vector or shRNA retrovirus. (F–G) Heatmaps showing genes rescued by *Pdk1* knockdown in pTH17 cells. WT and Mut pTH17 cells were independently transduced with *Pdk1* shRNA-expressing retrovirus (or control), and the expression of pathogenic signature genes (F) and metabolism-associated enzyme genes (G) is shown. Statistical significance was determined using one-way ANOVA. *n* = biological replicates as indicated. *p < 0.05, **p < 0.01, ***p < 0.001, and ****p < 0.0001. ns, not significant. All data are reported as mean ± SD, *n* = 3 biological replicates per group. See also Figure S7.

To further define how the enhanced RORγt^N275D^-HIF-1α interaction amplifies Th17 inflammatory potential, we analyzed the HIF-1α downstream genes that were altered in N275D-expressing Th17 cells (Figures 7A and S7A). Among these candidates, analysis of publicly available chromatin immunoprecipitation sequencing (ChIP-seq) datasets revealed overlapping RORγt and HIF-1α peaks at the *Pdk1* and *Ifngr1* loci (Figures 7B and 7C). Targeted ChIP assays confirmed enhanced recruitment of RORγt^N275D^ to these regions compared with WT RORγt (Figure 7D). These data indicate that the N275D mutation strengthens the RORγt-HIF-1α transcriptional axis at specific effector gene loci.

Among the affected targets, *Pdk1*, a well-established direct HIF-1α target,^31,46–48^ was significantly upregulated in RORγt^N275D^ cells (Figure 7A). PDK1 has been reported to trigger both mTOR signaling and HIF-1α signaling, positioning it as a potential integrator of metabolic and inflammatory pathways downstream of RORγt-HIF-1α cooperation.

To test the functional relevance of PDK1 induction, we knocked down *Pdk1* in pathogenic Th17 cells from WT and N275D mice using retroviruses expressing a pool of two independent short hairpin RNAs (shRNAs) to maximize efficiency and minimize off-target effects (Figures S7B and S7C). Silencing *Pdk1* markedly reduced the elevated frequencies of IL-17A^+^IFN-γ^+^ DP cells as well as IFN-γ-SP Th17 cells, characteristic of RORγt^N275D^ Th17 cells, restoring these populations to near WT levels (Figure 7E). In addition, silencing *Pdk1* broadly attenuated proinflammatory and metabolic gene expression programs (Figures 7F, 7G, S7E, and S7F). Together, these results establish *Pdk1* as a transcriptionally induced and functionally essential mediator of the heightened inflammatory phenotype driven by the RORγt^N275D^ mutation.^49^ Thus, N275D strengthens the RORγt-HIF-1α axis, directly boosting transcription of *Pdk1* and linking hypoxia signaling with Th17 effector function.

In summary, through systematic variant screening, we identified RORγt^N275D^ as a naturally occurring hyperactive allele. In KI mice, this mutation preserved normal T cell development and immune homeostasis but conferred heightened Th17 pathogenicity in colitis, with a clear gene dosage effect. Mechanistically, RORγt^N275D^ rewired the Th17 transcriptome and metabolome toward a hypoxia-associated inflammatory state by stabilizing RORγt-HIF-1α interactions and activating the downstream targets, such as *Pdk1*. These findings demonstrate how a single point mutation can amplify Th17-driven inflammation and provide mechanistic insight into genetic contributions to autoimmune disease.

## DISCUSSION

The *RORC* locus is strongly associated with autoimmune diseases such as IBD, multiple sclerosis, and psoriasis, yet the functional impact of individual variants has remained uncertain. Using genetic mouse models, transcriptomics, metabolomics, and functional assays, we identified N277D as a bona fide hyperactive allele, providing a framework for mechanistic analysis of *RORC* variants flagged in GWAS. This mutation carries three major clinical implications: (1) it establishes *RORC* mutations as modifiers of Th17 pathogenicity, supporting genetic screening in patients with early onset or treatment-refractory disease; (2) it demonstrates that heterozygous variants can be pathogenic, underscoring the importance of evaluating partial gain-of-function alleles; and (3) it positions the RORγt-HIF-1α-metabolism axis as a therapeutic target, where RORγt inhibition, disruption of its HIF-1α interaction, or blockade of downstream metabolic enzymes could benefit genetically susceptible patients. In addition to N277D, our screening assay identified several other RORγt variants (Q263H, K288Q, Q306H, and Q326K) that similarly enhanced IFN-γ production; while these variants are also of significant interest, their mechanistic and pathological effects will be addressed in future studies.

HIF-1α has long been recognized as a key driver of Th17 differentiation through induction of *Rorc* and repression of *Foxp3*. Our findings extend this paradigm by revealing a reciprocal regulatory axis in which RORγt amplifies HIF-1α transcriptional activity in a context-dependent manner. Notably, the N275D mutation does not perturb Th17 cell homeostasis under steady-state conditions but instead selectively enhances Th17 inflammatory potential during inflammation. This specificity likely reflects the inflammatory microenvironment, where increased glycolytic flux, metabolic stress, and hypoxia promote HIF-1α accumulation and activity. Under these conditions, the enhanced affinity of RORγt-N275D for HIF-1α becomes functionally consequential, driving exaggerated transcriptional responses rather than altering basal lineage commitment.

Mechanistically, the N275D mutation strengthens the physical interaction between RORγt and HIF-1α, leading to increased expression of canonical HIF-1α target genes, including *Pdk1*, and activation of glycolytic and hypoxia-responsive pathways. In addition to augmenting transcriptional output, this enhanced interaction modestly but significantly increases HIF-1α protein stability, suggesting that RORγt may partially shield HIF-1α from degradation. Given that ubiquitination of the HIF-1α has been implicated in proteasomal turnover, increased RORγt binding at bHLH may reduce accessibility to the degradation machinery, thereby prolonging HIF-1α signaling during inflammation.

Functionally, the reinforced RORγt-HIF-1α axis drives metabolic reprogramming in Th17 cells, as evidenced by reduced oxidative phosphorylation and a shift toward a glycolytic metabolic state. This decrease in OCR is consistent with heightened HIF-1α activity and supports a metabolic program optimized for effector function in hypoxic and nutrient-limited tissues. PDK1, as a direct HIF-1α target, further reinforces this state by promoting HIF-1α signaling, establishing a feedforward circuit that tightly couples transcriptional regulation with metabolic adaptation. These mechanisms are particularly relevant in metabolically stressed and hypoxic environments such as the inflamed intestinal mucosa, where sustained Th17 pathogenicity contributes to chronic inflammation. While PDK1 appears to be a direct and central effector of this pathway, broader HIF-1α-dependent metabolic remodeling likely also contributes to the enhanced effector function observed in mutant RORγt-expressing Th17 cells.

These findings establish RORγt as more than a lineage-specifying transcription factor. It is also a metabolic integrator that engages partners like HIF-1α to adapt Th17 responses to environmental conditions. The N275D mutation strengthens this partnership, driving transcriptional and metabolic programs that enhance effector cytokine production and colitogenicity. This mechanistic link explains the heightened inflammatory potential of mutant Th17 cells and illustrates how transcriptional and metabolic programs are inseparably intertwined in immune pathology.

Our results further indicate that RORγt activity is tunable by subtle structural changes. The enhanced HIF-1α binding conferred by N275D likely reflects a conformational shift in the LBD that alters cofactor recruitment. Consistent with prior studies implicating PTMs such as phosphorylation and ubiquitination,^17–20^ these findings highlight the dynamic regulation of RORγt. It also raises the possibility that naturally occurring variants or small molecules can selectively modulate RORγt partner interactions and metabolic status, with broad consequences for immune balance and disease susceptibility.

In summary, we uncover a mechanism by which a single amino acid substitution in RORγt amplifies Th17-mediated inflammation through strengthened cooperation with HIF-1α. This interaction integrates lineage-defining transcriptional programs with metabolic adaptation, showing how genetic variants can rewire immune function across multiple layers. By bridging genetic risk, mechanistic insight, and therapeutic opportunity, our work provides a framework for translating autoimmune-associated *RORC* variants into actionable biology. Targeting the RORγt-HIF-1α axis may enable precision therapies for Th17-driven diseases in genetically at-risk individuals.

### Limitations of the study

First, RORγt also governs ILC3 development and function. Although the N275D mutation did not affect ILC3 numbers or cytokine production under homeostatic conditions, its impact during inflammation remains unknown. Given the shared reliance of Th17 cells and ILC3s on RORγt- and HIF-1α-dependent programs, it is possible that N275D similarly enhances ILC3 effector function in inflammatory settings, which warrants future investigation. Second, this study lacks patient-derived data. The N275D variant is rare (population frequencies: 6.85e-7) in the human population and is unlikely to represent a common disease-causing allele; rather, it serves as a naturally occurring proof-of-concept variant that reveals a regulatory mechanism linking RORγt, metabolic reprogramming, and Th17 pathogenicity. Future studies will be needed to assess whether similar mechanisms operate in human inflammatory disease.

## RESOURCE AVAILABILITY

### Lead contact

Requests for further information and resources should be directed to and will be fulfilled by the lead contact, Zhiheng He (zhihengh@usc.edu).

### Materials availability

All unique/stable reagents generated in this study are available from the lead contact, with a completed materials transfer agreement.

### Data and code availability

- The RNA-seq data have been deposited at Gene Expression Omnibus (GEO) and are publicly available as of the date of publication. Accession numbers are listed in the key resources table. Data reported in this paper will be shared by the lead contact upon request.
- This paper does not report original code.
- This paper analyzes existing, publicly available data, accessible at GEO or ENCODE. Accession numbers are listed in the key resources table.
- Any additional information required to reanalyze the data reported in this paper is available from the lead contact upon request.

## STAR★METHODS

### EXPERIMENTAL MODEL AND STUDY PARTICIPANT DETAILS

#### Mice and ethics statement

Knockin RORγt^N275D^ mice (C57BL/6 background) were kindly provided by Dr. Zuoming Sun (City of Hope). Wild-type C57BL/6 (Stock No. 005304) and *Rag1*^−/−^ (Stock No. 002216) mice on a C57BL/6J background were purchased from the Jackson Laboratory. All mice were maintained under specific pathogen-free (SPF) conditions in the vivarium at the Keck School of Medicine of the University of Southern California (USC). Unless otherwise noted, experiments were performed using 6–12-week-old mice. Both male and female mice were included in all experiments, with age- and sex-matched groups and an equal sex distribution (1:1 ratio) whenever possible. A minimum of five animals per experimental condition were used. No sex-specific differences were observed in treatment responses; therefore, data from male and female mice were combined for analysis. For adoptive transfer colitis experiments, naive CD4^+^ T cells were isolated from wild-type or RORγt^N275D^ knock-in mice (homozygous RORγt^N275D/N275D^ or heterozygous RORγt^N275D/+^), using littermate controls when applicable, and transferred into age- and sex-matched *Rag1*^−/−^ recipient mice on the same C57BL/6J background. Donor and recipient mice were maintained under identical SPF conditions. All animal procedures were conducted in accordance with institutional guidelines and were approved by the USC Institutional Animal Care and Use Committee (IACUC).

### METHOD DETAILS

#### Plasmids and retrovirus

Mutant RORγt constructs were generated by PCR-based site-directed mutagenesis using a kit from Vazyme. MSCV-LTRmiR30-PIG (LMP) vector was purchased from Open Biosystems, and all shRNA plasmids were designed and assembled according to the vendor’s miR30-based protocol, including mPdk1-1 (TGCTGTTGACAGTGAGCGagctgagtatttctttcaagttTAGTGAAGCCACAGATGTAaacttgaaagaaatactcagcgTGCCTACTGCCTCGGA) and mPdk1-2 (TGCTGTTGACAGTGAGCGagccgaaagacatgaccacattTAGTGAAGCCACAGATGTAaatgtggtcatgtctttcggcgTGCCTACTGCCTCGGA). Retroviruses were produced in Platinum-Eco packaging cells (Cell Biolabs) by transfecting the expression plasmids with BioT (Bioland LLC), and viral supernatants were harvested at 48 h and 72 h, filtered through 0.4-μm filters, and stored at −80°C until use.

#### T cell development assay

FACS-sorted double-negative thymocytes (Thy1.2^+^ CD4^−^ CD8^−^) from *Rorc*^−/−^ mouse were seeded at 2 × 10^5^ cells/mL onto 80% confluent OP9-DL4 stromal monolayers in flat-bottom 24-well plates. Cultures were maintained in αMEM (Gibco) supplemented with 20% FBS, 100 U/ml penicillin–streptomycin, 2 mM L-glutamine (Invitrogen), and 5 ng/mL recombinant mouse IL-7 (Biolegend) overnight. The next day, co-cultures were transduced by spin infection (1,250 g, 30°C, 1.5 h) with retroviral supernatants in the presence of polybrene (8 mg/mL; Sigma-Aldrich). Following centrifugation, viral supernatant was replaced with fresh medium containing 5 ng/mL IL-7. Co-cultures were harvested 72 h post-transduction for flow-cytometric analysis.

#### Naive CD4^+^ T cell isolation and *in vitro* Th17 differentiation

Naive CD4^+^ T cells were isolated by negative selection using the Mouse CD4^+^ T cell Isolation Kit (Miltenyi Biotec). Cells were resuspended at 4×10^5^ cells/ml in Iscove’s modified DMEM (Corning) supplemented with 2 mM L-glutamine, 50 mM 2-mercaptoethanol, 100 U/ml penicillin, 100 mg/mL streptomycin, and 10% FBS, and cultured for 3 days in 24-well plates pre-coated with goat anti-hamster IgG (1 mg/mL; Jackson ImmunoResearch Labs) in the presence of anti-CD3 (145–2C11; 0.125 μg/mL; Biolegend) and anti-CD28 (37.51; 1 μg/mL; Biolegend). For polarization, conventional Th17 conditions included TGF-β (4 ng/mL; Biolegend), IL-6 (20 ng/mL; Biolegend), anti-IL-4 (11B11; 5 μg/mL), and anti-IFN-γ (XMG1.2; 5 μg/mL); pathogenic Th17 conditions further included IL-23 (20 ng/mL; Biolegend) and IL-1β (20 ng/mL; Biolegend). Th1 differentiation used IL-12 (20 ng/mL; IL-23 (20 ng/mL; Biolegend)) plus anti-IL-4 (5 μg/mL); and Treg differentiation used TGF-β (5 ng/mL). For retroviral transduction, T cells were pre-activated for 24 h with anti-CD3/anti-CD28 on goat anti-hamster–coated plates, then spin-infected with retroviral supernatants (1,250 g, 30°C, 1.5 h) in the presence of polybrene (8 mg/mL; Sigma-Aldrich); after infection, viral supernatants were replaced with cytokine-containing media to continue *in vitro* differentiation.

#### Transfer colitis induction

Spleens from 8 to 10-week-old donor mice (WT, RORγt^N275D^ heterozygous or RORγt^N275D^ homozygous) were mechanically dissociated through a 40-μm strainer to generate single-cell suspensions. Naive CD4^+^ T cells were enriched by negative magnetic selection using the Mouse CD4^+^ T cell Isolation Kit (Miltenyi Biotec). Cells were resuspended in PBS and transferred intraperitoneally into 7–9-week-old *Rag1*^−/−^ recipients at 1×10^6^ cells per 100 μL. Body weight and clinical signs of colitis were monitored over the course of the model, and humane endpoints typically occurred 4–6 weeks after transfer.

#### T cell isolation from colon and small intestine

Colon was collected at the endpoint of transfer colitis and small intestine was collected from mice of age 6–8 weeks, luminal contents were flushed with PBS, tissues were everted, cut into ~1-cm segments, and incubated in an epithelial-stripping buffer (5 mM EDTA and 145 μg/ml dithiothreitol in DMEM) at 37°C with shaking (200 rpm) for 20 min. Samples were vortexed, rinsed in PBS to remove EDTA and epithelium, minced, and digested in collagenase D (1 mg/mL) and DNase I (100 μg/mL) at 37°C for 40 min with shaking (200 rpm). Cell suspensions were triturated, passed through a 70-μm strainer, resuspended in 40% Percoll, overlaid onto 80% Percoll, and centrifuged at 500 × g for 25 min with no brake. Lymphocytes were collected from the interphase, washed, and processed for downstream analyses.

#### Flow cytometry and cytokine staining

*In vitro*–activated or freshly isolated primary cells were restimulated for 2 h at 37°C with PMA (20 ng/mL), ionomycin (1 μg/mL), and brefeldin A (BFA; 5 μg/mL; Biolegend). Cells were labeled with a fixable viability dye (Zombie Aqua, BioLegend), stained for surface markers, and then fixed/permeabilized for intracellular cytokine staining using the eBioscience Foxp3/Transcription Factor Staining Buffer set with antibodies listed in Table S1. Samples were acquired on a BD FACSConto and analyzed in FlowJo (v10.10.0). Th17 cells were identified as live CD45^+^ CD3ε^+^ CD4^+^ RORγt^+^ events.

#### RNA sequencing (RNA-seq) and analysis

Naive CD4^+^ T cells from WT and RORγt^N275D^ mice and polarized under pathogenic Th17 conditions for 3 days prior to RNA isolation (Qiagen). Library preparation and sequencing were performed as DNBSEQ eukaryotic strand-specific mRNA libraries; six libraries in total were sequenced on the DNBSEQ platform with paired-end 150-bp reads (PE150) and Phred+33 quality encoding. Reads were quality-checked and trimmed, aligned to the mouse genome (mm10) with STAR, and counted with featureCounts. Differential expression was computed using DESeq2; genes with FDR-adjusted *p* < 0.05 and |log_2_ (fold change) | ≥ 1 were called significant. Heat maps display gene-wise z-scored expression values and hierarchical clustering was performed in R (pheatmap).

#### Metabolite profiling

Th17 cells were generated *in vitro* (see “*In vitro* Th17 differentiation”) with. For each sample, 5 × 10^6^ cells were collected. Intracellular metabolites were extracted by resuspending the cell pellet in 1 mL of 80% methanol prechilled to −80°C, incubating at −80°C for 20 min, and pelleting at 4°C, 15,000 rpm, 10 min. The supernatant was saved; the pellet was re-extracted with 200 μL ice-cold 80% methanol, centrifuged again, and supernatants were combined. Extracts were dried under vacuum at room temperature and reconstituted in water for LC–MS.

#### Quantitative PCR (qPCR)

Total RNA was purified and reverse-transcribed to cDNA, which served as template for quantitative PCR using SYBR Green PCR Master Mix on an Biorad CFX Duet Real-Time system with primers listed in Table S2. Relative transcript abundance was calculated by the 2 ^–ΔΔCt method after normalization to β-actin (Actb). Primer sets were validated for optimal amplification efficiency, and all targets were measured in technical duplicates across two independent experiments.

#### Western blot and immunoprecipitation

Cells were lysed in 1% Triton X-100 buffer (50 mM Tris-HCl, pH 7.4, 150 mM NaCl, 5 mM EDTA, 1X Protease Inhibitor, 1 mM PMSF). Equal amounts of protein from cell lysates were separated on SDS-PAGE, transferred to PVDF membranes (Millipore), and detected with the indicated primary antibodies. For immunoprecipitation, cell lysis was incubated overnight at 4°C with the relevant antibody and another protein A/G beads incubation for 2 h, washed four times in lysis buffer, and the bound material was eluted, resolved by SDS-PAGE, and analyzed by immunoblotting.

#### Chromatin immunoprecipitation (ChIP)

2×10^7^ cells were cross-linked with 1% formaldehyde for 5 min at room temperature and stopped with 125 mM glycine for 5 min. Nuclei was prepared and chromatin was isolated using the Active Motif Enzymatic Shearing kit per manufacturer’s instructions. Briefly, cells were lysed in the kit lysis buffer, nuclei were pelleted and resuspended in shearing buffer, and chromatin was digested with the Enzymatic Shearing Cocktail at 37°C to ~200–500 bp 15 min. The reaction was stopped with EDTA, and lysates were clarified by centrifugation (12,000 × g, 10 min, 4°C). Equal amounts of sheared chromatin were incubated overnight at 4°C with anti-RORγt (BD Biosciences, clone Q31–378) or isotype-matched IgG control, followed by capture with magnetic protein A/G beads for 2 h. Beads were washed sequentially with the kit low-salt, high-salt and final wash buffers. Complexes were eluted in the kit elution buffer and cross-links were reversed at 65°C 4 h and Proteinase K treatment. DNA was purified using the kit spin columns and used for ChIP-seq library preparation or ChIP-qPCR quantification of specific loci.

#### Tissue preparation and H&E staining

Colons were harvested and longitudinally opened to expose the epithelial surface. Tissues were thoroughly rinsed with cold PBS to remove fecal contents and mucus, followed by a brief rinse in 10% neutral buffered formalin. The colons were then rolled into Swiss rolls using a pipette tip, placed into tissue cassettes, and fixed overnight in 10% neutral buffered formalin. Fixed tissues were paraffin-embedded, sectioned to include the full length of the colon, and mounted onto glass slides. Sections were stained with hematoxylin and eosin (H&E) according to standard protocols. Representative images were acquired using a Nikon Eclipse TE300 inverted microscope. Histopathological evaluation of inflammation and tissue damage was performed by a trained pathologist in a blinded manner (see Histology scoring).

#### Histopathological scoring

Histopathological evaluation of H&E-stained colonic sections was performed by a trained pathologist according to established criteria as previously described. Tissue damage and inflammation were assessed based on the following parameters: epithelial hyperplasia (percentage increase compared with control tissue; 0 = no change, 1 = 1–50%, 2 = 51–100%, 3 = >100%); epithelial integrity (0 = no change, 1 = <10 epithelial cells shed per lesion, 2 = 11–20 cells shed per lesion, 3 = epithelial ulceration, 4 = severe crypt destruction); infiltration of granulocytes and mononuclear cells (0 = none, 1 = mild, 2 = moderate, 3 = severe); goblet cell depletion (evaluated under ×400 magnification; 0 = >50 cells per high-power field [HPF], 1 = 25–50/HPF, 2 = 10–25/HPF, 3 = <10/HPF); and submucosal edema (0 = none, 1 = mild, 2 = moderate, 3 = severe). The final histopathological score was calculated as the sum of all individual parameters.

#### Bioenergetic assays

Real-time oxygen consumption rate (OCR) was measured using a Seahorse Mini HS XF Analyzer (Agilent). Pathogenic Th17 cells were generated *in vitro* according to the indicated experimental conditions before. Briefly, 7 × 10^5^ *in vitro*-differentiated pathogenic Th17 cells were plated per well onto Seahorse XFp PDL-coated cell culture miniplates in triplicate, using Seahorse XF base medium (Agilent) supplemented with 1 mM sodium pyruvate, 2 mM L-glutamine, and 10 mM glucose, and lacking phenol red and serum. A T cell metabolic profiling assay (Agilent) was performed according to the manufacturer’s instructions. Following baseline measurements, 1.5 μM oligomycin, 2.5 μM BAM15, and 0.5 μM rotenone/antimycin were sequentially injected to assess mitochondrial function, with each compound loaded into the appropriate port of the sensor cartridge to achieve the indicated final concentrations during the assay.

### QUANTIFICATION AND STATISTICAL ANALYSIS

All statistical analyses are performed using GraphPad Prism (GraphPad Software) and, where indicated, Microsoft Excel. Details of statistical tests, exact values of *n*, definitions of *n*, and measures of center and dispersion are reported in the figure legends and, where appropriate, within the figures themselves. Unless otherwise stated, *n* represents the number of biologically independent samples, mice, or independent experiments, as specified in each figure legend. Data are generally presented as mean ± SEM unless otherwise noted. Statistical significance is assessed using unpaired two-tailed Student’s *t* tests for comparisons between two groups or one-way or two-way ANOVA with appropriate post hoc multiple-comparison tests for experiments involving more than two groups, as indicated in the figure legends. For mouse colitis experiments, mice are randomly assigned to experimental groups when possible, and no animals are excluded from analysis unless predefined humane endpoints are reached or technical failures occur, in which case exclusions are noted. A *p*value < 0.05 is considered statistically significant, and exact *p* values or significance levels are reported in the figures or figure legends.

## Supplementary Material

Supplementary data

Supplemental information can be found online at https://doi.org/10.1016/j.celrep.2026.117123.

## Figures and Tables

**Table:** KEY RESOURCES TABLE

REAGENT or RESOURCE	SOURCE	IDENTIFIER
Antibodies		
PE/Cyanine5 anti-mouse CD45 Antibody, clone 30-F11	Biolegend	RRID AB_312974 (Cat. No. 103109)
FITC anti-mouse CD45 Antibody, clone 30-F11	Biolegend	RRID AB_312972 (Cat. No. 103107)
FITC anti-mouse CD4 Antibody, clone GK1.5	Biolegend	RRID AB_312713 (Cat. No. 100510)
PE/Cyanine7 anti-mouse CD4 Antibody, clone RM4-5	Biolegend	RRID AB_11203903 (Cat. No. 304229)
PerCP/Cyanine5.5 anti-mouse CD4 Antibody, clone M4-5	Biolegend	RRID AB_893324 (Cat. No. 100434)
Pacific Blue^™^ anti-mouse CD4 Antibody, clone GK1.5	Biolegend	RRID AB_493647 (Cat. No. 100428)
APC-eFluor^™^ 780 anti-mouse CD3 Monoclonal Antibody, clone 17A2	eBioscience	RRID AB_1272181 (Cat. No.47-0032-82)
PE anti-mouse TCR β chain Antibody, clone H57-597	Biolegend	RRID AB_313430 (Cat. No. 109207)
PerCP-Cyanine5.5 anti-mouse CD8a Monoclonal Antibody, clone 53-6.7	eBioscience	RRID AB_2921016 (Cat. No. 45-0081-82)
Brilliant Violet^™^ 421 anti-mouse CD8a Monoclonal Antibody, clone 53-6.7	eBioscience	RRID AB_2921016 (Cat. No. 404-0081-80)
APC anti-mouse/human CD44 Antibody, clone IM7	Biolegend	RRID AB_312962 (Cat. No. 103011)
FITC anti-mouse CD25 Antibody, clone 3C7	Biolegend	RRID AB_961210 (Cat. No. 101907)
PE/Cyanine7 anti-mouse CD24 Antibody, clone 30-F1	Biolegend	RRID AB_2819878 (Cat. No. 138507)
Brilliant Violet 421^™^ anti-mouse CD5 Antibody, clone 53-7.3	Biolegend	RRID AB_2562173 (Cat. No. 100617)
APC anti-mouse CD90.2 (Thy1.2) Antibody, clone 53-2.1	Biolegend	RRID AB_10645337 (Cat. No. 140311)
PE Mouse anti-Mouse RORγt, clone Q31-378	BD Biosciences	RRID:AB_11153137 (Cat. No. 562607)
Brilliant Violet 421^™^ anti-mouse FOXP3 Antibody, clone MF-14	Biolegend	RRID AB_2565933 (Cat. No. 126419)
APC anti-T-bet Antibody, clone 4B10	Biolegend	RRID AB_10896913 (Cat. No. 644813)
APC anti-mouse IL-17A Antibody, clone TC11-18H10.1	Biolegend	RRID AB_536017 (Cat. No. 506915)
PE-Cyanine7 anti-mouse IFN gamma Monoclonal Antibody, clone XMG1.2	eBioscience	RRID AB_469680 (Cat. No. 25-7311-82)
Spark UV^™^ 387 anti-human/mouse Granzyme B Recombinant Antibody, clone QA18A28	Biolegend	RRID AB_3662295 (Cat. No. 396441)
Brilliant Violet 605^™^ anti-mouse TNF-α Antibody, clone MP6-XT22	Biolegend	RRID AB_11123912 (Cat. No. 506329)
Brilliant Violet 650^™^ anti-mouse IL-10 Antibody, clone JES5-16E3	Biolegend	RRID AB_3097267 (Cat. No. 505039)
Alexa Fluor^®^ 488 anti-mouse IL-17F Antibody, clone 9D3.1C8	Biolegend	RRID AB_10660963 (Cat. No. 517005)
APC Granzyme A Monoclonal Antibody, clone GzA-3G8.5	eBioscience	RRID AB_2573228 (Cat No.17-5831-82)
PerCP/Cyanine5.5 anti-mouse IL-22 Antibody, clone Poly5164	Biolegend	RRID AB_2563373 (Cat. No. 516411)
Spark Blue^™^ 574 anti-mouse CD4 Antibody, clone GK1.5	Biolegend	RRID AB_2904268 (Cat. No. 100489)
Alexa Fluor^®^ 700 anti-mouse CD45 Antibody, clone 30-F11	Biolegend	RRID AB_493714 (Cat. No. 103127)
PE anti-human HIF1α Antibody, clone 546-16	Biolegend	RRID AB_2562422 (Cat. No. 359703)
Monoclonal ANTI-FLAG antibody, clone M2	Sigma	RRID:AB_259529 (Cat. No. F3165)
Monoclonal Anti-HA antibody, clone HA-7	Sigma	RRID AB_262051 (Cat. No. H3663)
Purified Mouse Anti-RORγt, clone Q31-378	BD Biosciences	RRID AB_10894594 (Cat. No. 562197)
HIF-1 alpha Polyclonal antibody	Proteintech	RRID AB_10732601 (Cat. No. 20960-1-AP)
PDK1 Polyclonal antibody	Proteintech	RRID AB_10598310 (Cat. No. 18262-1-AP)
Anti-Actin antibody, clone 20–30	Sigma	RRID AB_476738 (Cat. No. A5060)
Ultra-LEAF^™^ Purified anti-mouse CD3 Antibody, clone 17A2	Biolegend	AB_2810313 (Cat. No. 100239)
Ultra-LEAF^™^ Purified anti-mouse CD28 Antibody, clone 37.51	Biolegend	RRID AB_11147170 (Cat. No. 102116)
InVivoMAb anti-mouse IFNγ, clone XMG1.2	bioXcell	RRID AB_1107694 (Cat. No. BE0055)
InVivoMAb anti-mouse IL-4, clone 11B11	bioXcell	RRID AB_1107707 (Cat. No. BE0045)
AffiniPure^®^ Goat Anti-Syrian Hamster IgG (H + L)	Jackson Immuno Research Labs	RRID AB_2337450 (Cat. No.107-005-142)
AffiniPure^®^ Donkey Anti-Mouse IgG (H + L) HRP	Jackson Immuno Research Labs	RRID AB_2340758 (Cat. No. 715-005-150)
AffiniPure^®^ Donkey Anti-Rabbit IgG (H + L) HRP	Jackson Immuno Research Labs	RRID AB_10015282 (Cat. No. 711-035-152)
Bacterial and virus strains		
NEB^®^ 5-alpha Competent *E. coli* (High Efficiency)	New England Biolabs	Cat #C2987H
Chemicals, peptides, and recombinant proteins		
Recombinant Mouse IL-6 (carrier-free)	Biolegend	Cat # 575702
Recombinant Mouse IL-1β (carrier-free)	Biolegend	Cat # 575102
Recombinant Mouse IL-23 (carrier-free)	Biolegend	Cat # 589002
Recombinant Mouse TGF-β1 (carrier-free)	Biolegend	Cat # 763102
Recombinant Mouse IL-7 (E. coli expressed, carrier-free)	Biolegend	Cat # 567204
Recombinant Mouse IL-12 (p70) (carrier-free)	Biolegend	Cat # 577002
Phorbol 12-myristate 13-acetate (PMA)	Sigma-Aldrich	Cat #P1585
Ionomycin	Sigma-Aldrich	Cat #I0634
Cycloheximide	Sigma-Aldrich	Cat # 239763
Brefeldin A Solution (1,000X)	Biolegend	Cat # 420601
Zombie Aqua^™^ Fixable Viability Kit	Biolegend	Cat # 423101
Collagenase from Clostridium histolyticum	Sigma-Aldrich	Cat #C2139
RNase-free DNaseI	Sigma-Aldrich	Cat # 4716728001
2-Mercaptoethanol (1000X)	Gibco	Cat # 21985-023
Polybrene	Sigma-Aldrich	Cat # TR1003G
Clarity ECL Western Blotting Substrates	Bio-rad	Cat # 170-5060
Precision Plus Protein Dual Color Standards	Bio-rad	Cat # 610374
Pierce^™^ Protease Inhibitor Mini Tablets, EDTA-free	Thermo Scientific	Cat # PIA32955
PVDF	Millipore sigma	Cat # IPVH00010
BamHI-HF	NEB	Cat #R3136L
XhoI	NEB	Cat #R0146L
EcoRI-HF	NEB	Cat #R3101L
SpeI-HF	NEB	Cat #R3133L
T4 DNA Ligase	NEB	Cat #M0202S
DPBS	Geneclone	Cat # 25-508
Percoll	Cytiva	Cat # 45-001-748
EDTA	Thermo Scientific	Cat #J15694-EA
DTT (Dithiothreitol)	Sigma-Aldrich	Cat # DTTRO
GeneRuler 1 kb DNA Ladder	Thermo Scientific	Cat # SM0311
DMEM	Corning	Cat # 10-013-CV
IMDM	Corning	Cat # 10-016-CV
MEM α	Gibco	Cat # 12561056
L-glutamine	Gibco	Cat # 25030081
Penicillin/streptomycin	Gibco	Cat # 15140122
BioT	Bioland Scientific LLC	Cat #B01-01
High-Capacity cDNA Reverse Transcription Kit	Applied Biosystems	Cat # 43-688-14
SYBR^™^ Green Universal Master Mix	Applied Biosystems	Cat # 4309155
2 × Phanta Flash Master Mix (Dye Plus)	Vayzme	Cat #P520-03
ClonExpress Ultra One Step Cloning Kit V2	Vayzme	Cat #C116-01
FBS	Gibco	Cat # A5256701
Critical commercial assays		
Naive CD4^+^ T cell Isolation Kit, mouse	Miltenyi Biotec	Cat # 130-104-453
Foxp3/Transcription Factor Staining Buffer Set	eBioscience	Cat # 00-5523-00
Seahorse XFp T cell Metabolic Profiling Kit	Agilent	Cat # 103771-100
Mut Express II Fast Mutagenesis Kit V2	Vayzme	Cat #C214
ChIP-IT^®^ Express Enzymatic Shearing Kit	Active motif	Cat # 53035
RNeasy Mini Kit	Qiagene	Cat # 74104
QIAquick Gel extraction kit	Qiagene	Cat # 28704
QIAprep Spin Miniprep Kit	Qiagene	Cat # 27104
Deposited data		
Pathogenic Th17 WT versus Mut RNA-seq	This paper	GSE307907
Experimental models: Cell lines		
HEK293 cells	ATCC	Cat # CRL-1573; RRID: CVCL_0045
PlatE cells	Cell Biolabs, Inc	Cat # RV-101
OP9-DL4	Sigma-Aldrich	Cat # SCC494
Experimental models: Organisms/strains		
C57BL/6NJ	Jackson Laboratories	Stock # 005304
*Rag1*^−/−^ (B6.129S7-*Rag1*^*tm1Mom*^/J)	Jackson Laboratories	Stock # 003145
B6.129P2-Rorctm1Litt/J	Jackson Laboratories	Stock # 007571
Knock-in RORγt N275D	Biocytogen	Project # EGE-LJL-080mKI
Oligonucleotides		
Primers for qRT-PCR, see Table S1	This paper	N/A
ChIP primers, see Table S1	This paper	N/A
Recombinant DNA		
MSCV	Addgene	Plasmid #24828
LMP	Open Biosystems	Cat # EAV4071
pMSCV-RORγt	Homemade	N/A
pMSCV-RORγt-Q263H	Homemade	N/A
pMSCV-RORγt-R265Q	Homemade	N/A
pMSCV-RORγt-R271W	Homemade	N/A
pMSCV-RORγt-N275D	Homemade	N/A
pMSCV-RORγt-R279P	Homemade	N/A
pMSCV-RORγt-K288Q	Homemade	N/A
pMSCV-RORγt-Q306H	Homemade	N/A
pMSCV-RORγt-Q362K	Homemade	N/A
pMSCV-RORγt-R344G	Homemade	N/A
pMSCV-RORγt-K358I	Homemade	N/A
pMSCV-RORγt-E383G	Homemade	N/A
pMSCV-RORγt-R407W	Homemade	N/A
pMSCV-RORγt-E412G	Homemade	N/A
pMSCV-RORγt-H429Q	Homemade	N/A
pMSCV-RORγt-H430L	Homemade	N/A
pMSCV-RORγt-K446N	Homemade	N/A
pMSCV-RORγt-H467Y	Homemade	N/A
pMSCV-RORγt-E488K	Homemade	N/A
pLVX-Hif1a-3flag	Homemade	N/A
pLVX-Hif1a-1-80aa-3flag	Homemade	N/A
pLVX-Hif1a-81-200aa-3flag	Homemade	N/A
pLVX-Hif1a-201-329aa-3flag	Homemade	N/A
pLVX-Hif1a-330-427aa-3flag	Homemade	N/A
pLVX-Hif1a-428-528aa-3flag	Homemade	N/A
pLVX-Hif1a-529-826aa-3flag	Homemade	N/A
pIP-RORγt-HA	Homemade	N/A
pLMP-shHif1a	Homemade	N/A
pLMP-shPdk1	Homemade	N/A
Software and algorithms		
GraphPad Prism	GraphPad Software	https://www.graphpad.com
FlowJo (Version 10.4.1)	TreeStar	https://www.flowjo.com
R studio	R studio	N/A
Deposited data		
Raw and analyzed sequencing data	This paper	GSE307907
ChIP-seq data (RORC, murine Th17)	Gene Expression Omnibus	GSM1004856
ChIP-seq data (HIF1A, murine Th17)	Gene Expression Omnibus	GSM1004819

## Highlights

- ROR**γ**t N277D/N275D exacerbates Th17 colitis without altering T cell homeostasis
- Mutation elevates IFN-**γ** and Th1 cytokines in Th17 cells via HIF-1**α**
- Multi-omics link glycolysis to HIF-1**α**/ROR**γ**t N275D binding at *Pdk1* locus
- *Pdk1* silencing normalizes IFN-**γ**, defining a ROR**γ**t-HIF-1**α**-PDK1 axis
