# Supplementary material for: Enhanced HIF-1α cooperation by a human RORγt mutant potentiates Th17 pathogenicity
Source: Cell Rep. Author manuscript; Available in PMC 2026 Sep 24. (PMC13603313; doi:10.1016/j.celrep.2026.117123)
Supplement: Supplementary file 1 [file NIHMS2208214-supplement-Supplementary_data.pdf]

Cell Reports, Volume *45*

Supplemental information

Enhanced HIF-1α cooperation by a human RORγt mutant potentiates Th17 pathogenicity

Peixian Dong, Liang Liu, John Yu, Ning Li, Benjamin Davidorf, Steven Shen, Ronald Bouch, Jing Zhang, David Ho, Gutian Xiao, Omid Akbari, Bing Zhang, and Zhiheng He

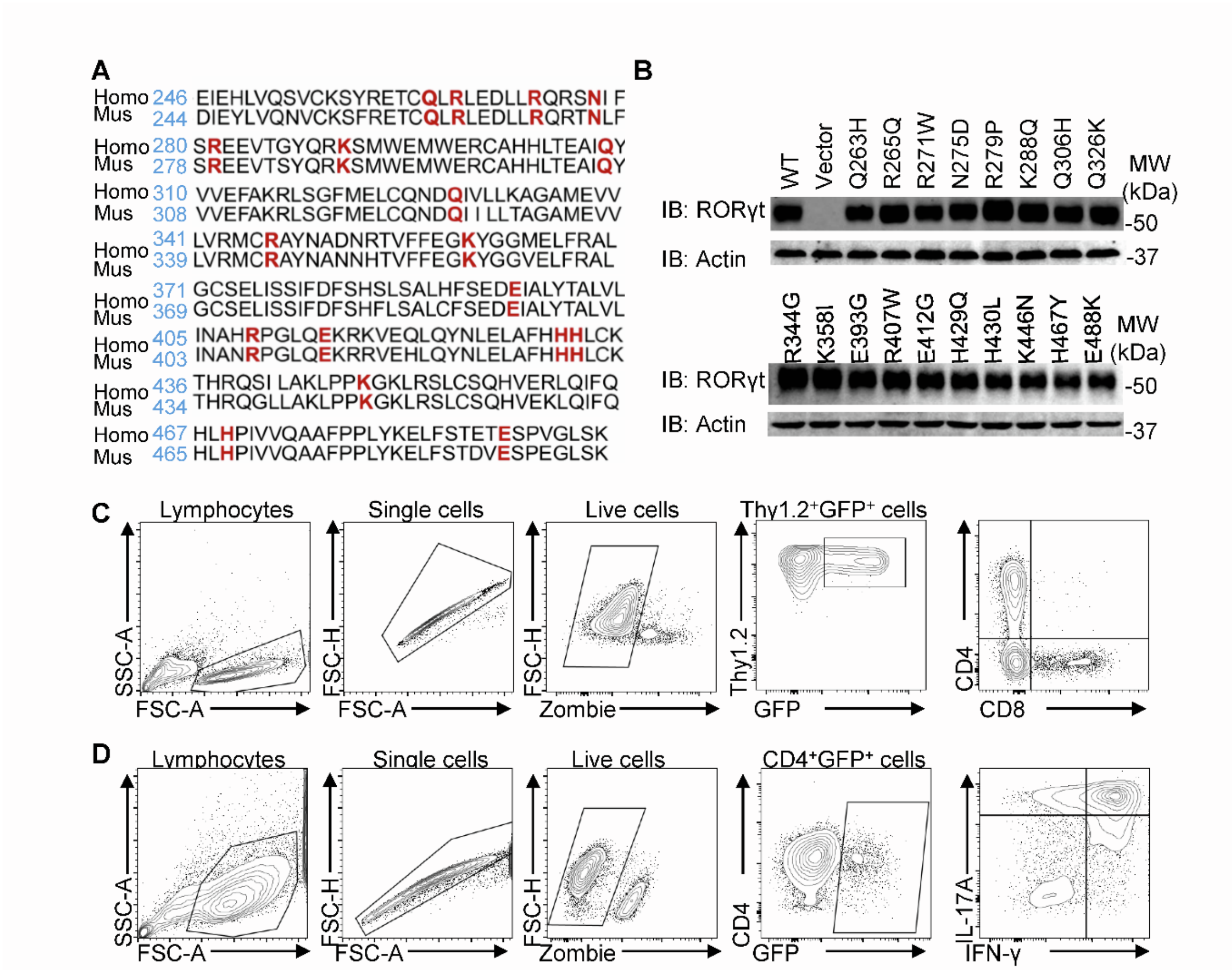

Figure S1. Validation of RORγt mutants in murine T cells, related to Figure 1.

(A) Human-mouse sequence alignment of the RORγt LBD highlighting conserved residues (red) targeted for mutagenesis. **(B)** Immunoblot validation of retroviral expression of WT and mutant RORγt in primary *Rorc*$^{-/-}$ T cells, showing comparable protein levels (RORγt, upper; Actin, lower). **(C)** Gating strategy for flow cytometric analysis of retrovirally transduced *Rorc*$^{-/-}$ progenitors. **(D)** Gating strategy for flow cytometric analysis of retrovirally transduced *Rorc*$^{-/-}$ pathogenic Th17 cells.

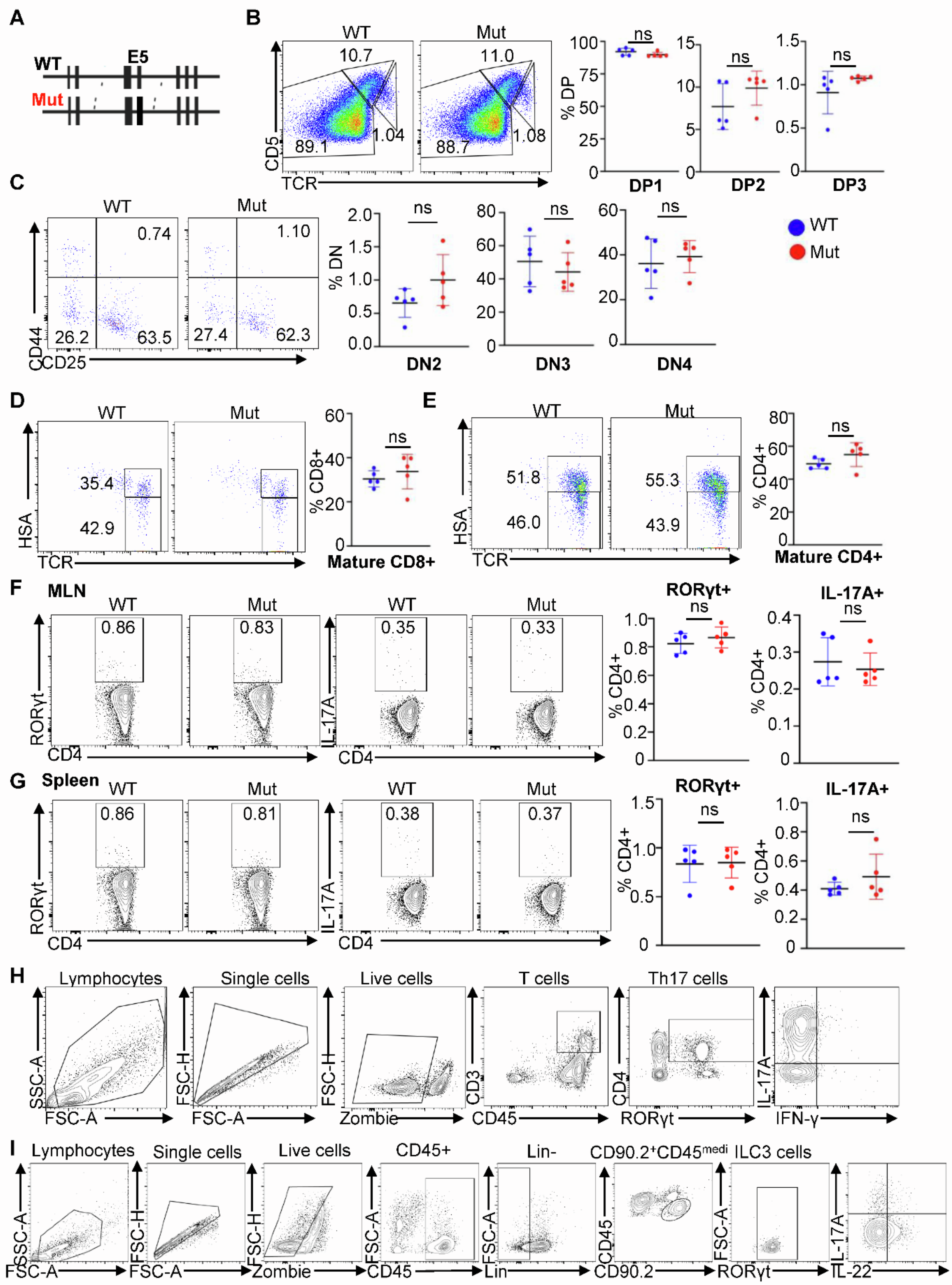

A
WT
E5
Mut
B
WT
Mut
10.7
11.0
89.1
1.04
88.7
1.08
CD5
TCR
% DP
DP1
DP2
DP3
ns
C
WT
Mut
0.74
1.10
26.2
63.5
27.4
62.3
CD44
CD25
% DN
DN2
DN3
DN4
WT
Mut
D
WT
Mut
35.4
42.9
HSA
TCR
% CD8+
Mature CD8+
E
WT
Mut
51.8
46.0
55.3
43.9
HSA
TCR
% CD4+
Mature CD4+
F
MLN
0.86
0.83
0.35
0.33
RORγt
IL-17A
CD4
RORγt+
IL-17A+
G
Spleen
0.86
0.81
0.38
0.37
H
Lymphocytes
Single cells
Live cells
T cells
Th17 cells
SSC-A
FSC-A
FSC-H
Zombie
CD3
CD45
IFN-γ
I
CD45+
Lin-
CD90.2+CD45medi
ILC3 cells
Lin
CD90.2
IL-22

Figure S2. Generation and characterization of RORγt^{N275D} knock-in mice, related to Figure 2.

(A) Schematic of the CRISPR/Cas9 knock-in strategy at the mouse *Rorc* locus introducing the N275D substitution in exon 5. **(B)** Flow cytometric analysis of $CD4^+CD8^+$ thymocytes from the indicated genotypes (left) with frequencies of DP1–DP3 subsets quantified (right). **(C)** Flow cytometric analysis of $CD4^-CD8^-$ thymocytes from the indicated genotypes (left) with frequencies of DN2–DN4 subsets quantified (right). **(D-E)** Flow cytometric analysis of $CD4^+$ and $CD8^+$ thymocytes (left) with frequencies of HSA^{high} mature $CD4^+$ and $CD8^+$ thymocytes quantified (right). (**F-G**) Flow cytometry analysis (left) of $ROR\gamma t^+$ cells from mesenteric lymph node (MLN) (F) and spleen (G); Statistics of frequencies (right) of IL-$17A^+$ cells. **(H)** Gating strategy for analysis of small-intestinal lamina propria homeostatic Th17 ($ROR\gamma t^+$) cells in WT and mutant mice. **(I)** Gating strategy for analysis of cytokine production by small-intestinal lamina propria homeostatic ILC3 cells in WT and mutant mice. Statistical significance was determined using unpaired Student's *t* test. ns, not significant; All data reported as mean ± SD, n = 5 biological replicates per group.

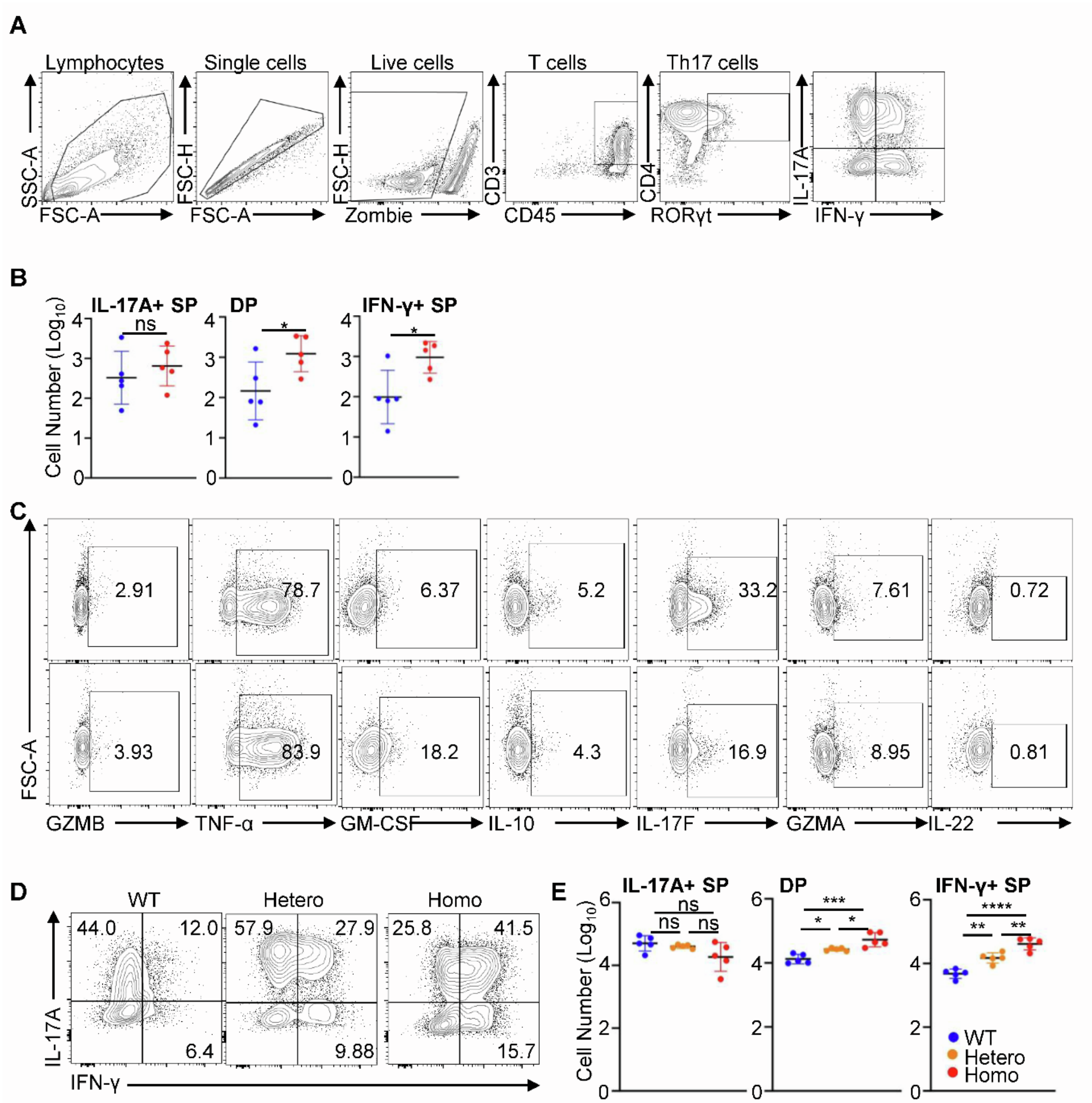

Figure S3. Enhanced pathogenicity of RORγt^{N275D} Th17 cells in transfer colitis, related to Figure 3.

(A) Gating strategy for flow cytometric analysis of lamina propria-infiltrating Th17 cells 6 weeks after transfer of WT, heterozygous, or homozygous RORγt^{N275D} T cells into *Rag1*$^{-/-}$ recipient mice. **(B)** Absolute numbers of IFN-γ$^+$ or IL-17A$^+$ Th17 cells in the lamina propria of transfer colitis recipients. **(C)** Representative flow cytometry plots of colonic GZMB$^+$, TNF-α$^+$, GM-CSF$^+$, IL-10$^+$, IL-17F$^+$, GZMA$^+$, IL-22$^+$ Th17 cells from transfer colitis recipients. **(D-E)** Representative flow cytometry plot of colonic IL-17A SP, DP and IFN-γ SP Th17 cells (left) and quantification of absolute numbers of IL-17A SP, DP and IFN-γ SP Th17 cells in the lamina propria of transfer colitis recipients. Statistical significance was determined by unpaired Student's *t*-test or one-way ANOVA. *, $P < 0.05$; **, $P < 0.01$; ****, $P < 0.0001$; ns, not significant. Data are presented as mean ± SD from $n = 5$ biological replicates per group.

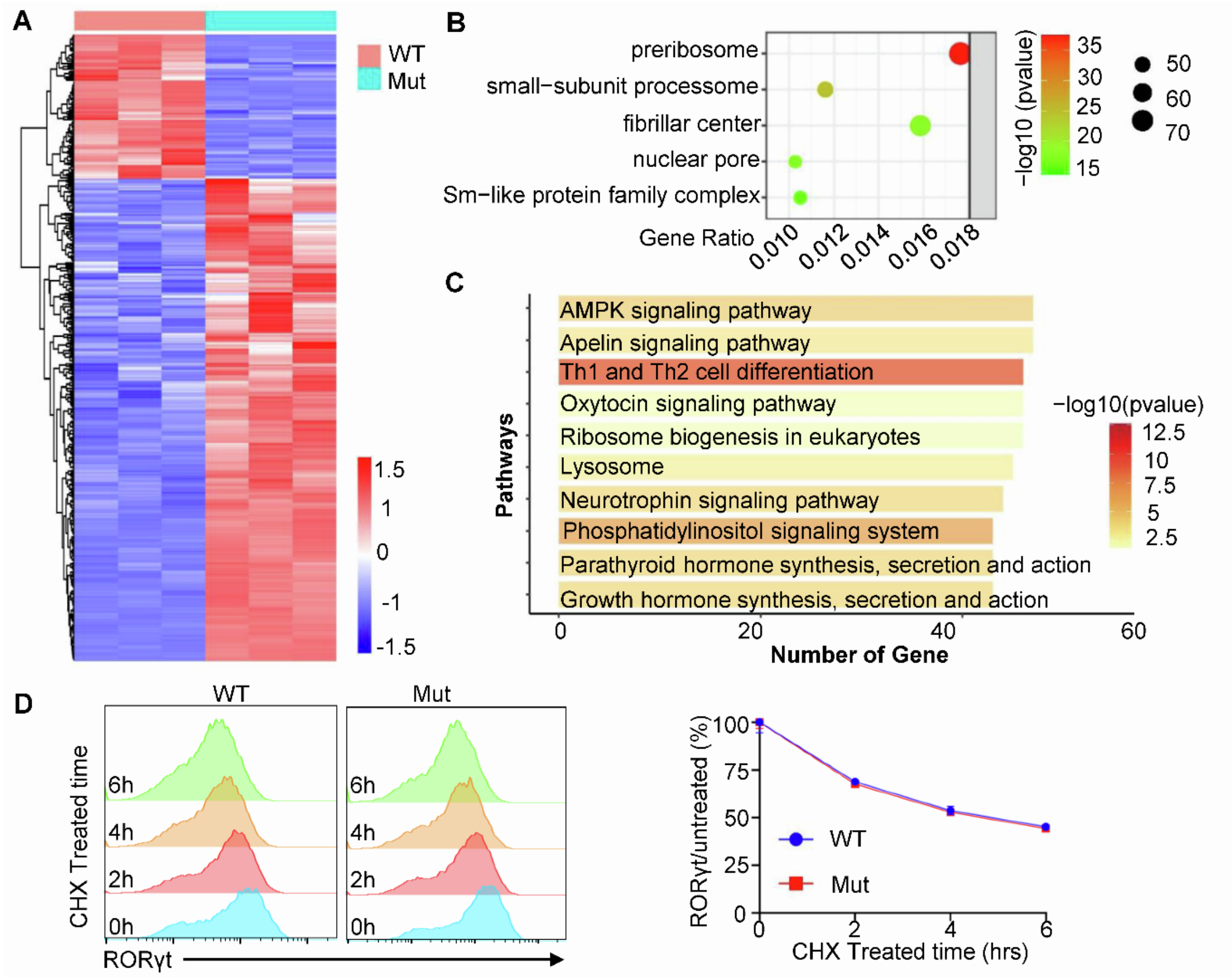

Figure S4. Transcriptomic profiling of RORγt^{N275D} Th17 cells and protein stability of RORγt^{N275D}, related to Figure 4.

(A) Heatmap of differentially expressed genes (DEGs) identified by RNA-seq in WT and mutant samples, with upregulated (red) and downregulated (blue) genes shown (row-scaled z-scores; columns, replicates; rows, genes). **(B)** Gene Ontology (GO) enrichment analysis of DEGs between WT and mutant groups. Bubble plots display Biological Process (top) and Molecular Function (bottom); dot size indicates gene count and color encodes significance (−log10 *p*-value). **(C)** KEGG pathway enrichment analysis of DEGs between WT and mutant groups. Bar length represents the number of genes per pathway, and color encodes significance (−log10 *p*-value). **(D)** Cycloheximide (CHX) chase assay of RORγt protein stability. Left, representative histograms of intracellular RORγt in WT and mutant cells at 0, 2, 4, and 6 h post-CHX treatment. Right, quantification of RORγt remaining (% of untreated) shows comparable decay kinetics between WT and mutant cells. Data are shown as mean ± SD from $n = 3$ biological replicates per group.

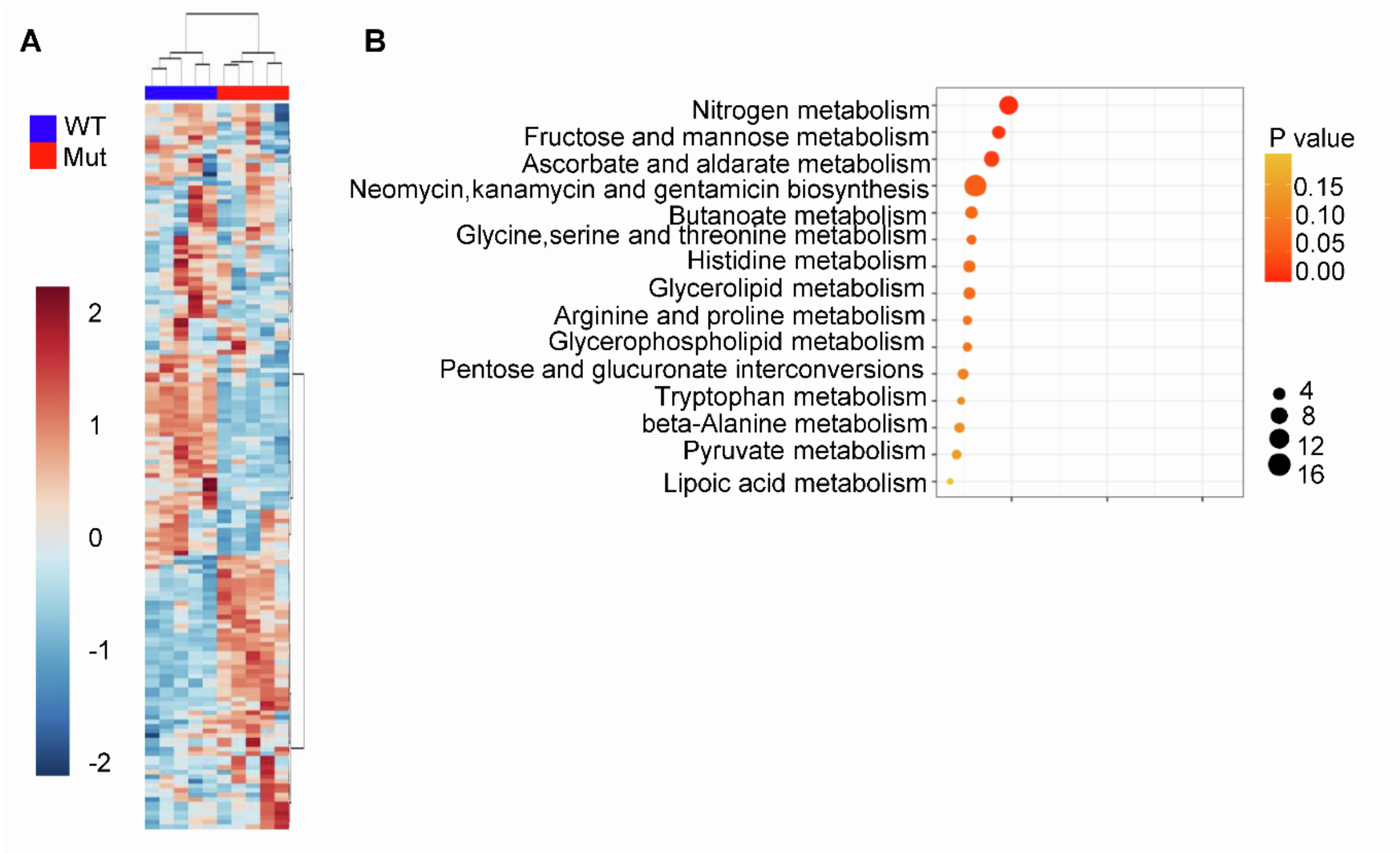

Figure S5. Metabolomic profiling of RORγt^{N275D} Th17 cells, related to Figure 5.

(A) Heatmap of global metabolomic profiling in WT and RORγt^{N275D} Th17 cells. Columns represent biological replicates, rows represent metabolites (row-scaled z-scores) clustered by similarity. Differentially abundant metabolites are listed. **(B)** Pathway enrichment analysis of significantly altered metabolic pathways. Dot size indicates the number of metabolites per pathway, and color encodes significance (−log10 *p*-value).

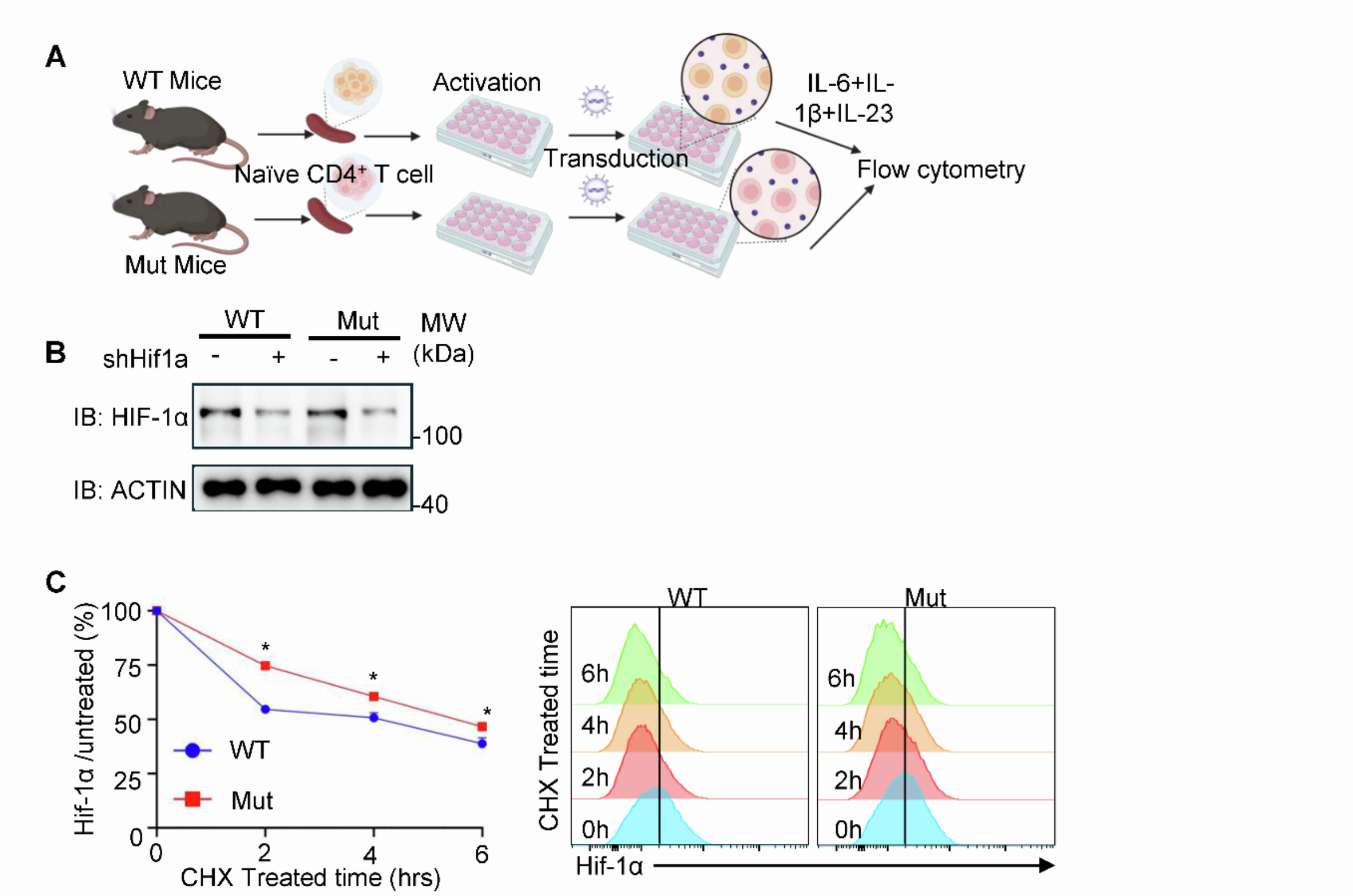

Figure S6. RORγt-N275D strengthens interaction with HIF-1α to drive pathogenicity, related to Figure 6.

(A) Experimental design of shRNA knocking down assays. WT or Mutant naïve CD4$^+$ T cells were seeded into anti-hamster coated flat-bottom 24-well plates, and then retrovirally transduced vector or shRNA followed pathogenic polarization condition (IL-6+IL-1β+IL-23) and flow cytometry. **(B)** Immunoblot validation of HIF-1α knockdown efficiency in Th17 cells transduced with vector or Hif1a shRNA retrovirus. MW, molecular weight (kDa). (**C**) CHX chase assay of RORγt protein stability. Left, representative histograms of intracellular HIF-1α in WT and mutant cells at 0, 2, 4, and 6 h post-CHX treatment. Right, quantification of HIF-1α remaining (% of untreated) shows comparable decay kinetics between WT and mutant cells. Statistical significance was determined using unpaired Student's *t* test. Data are shown as mean ± SD from *n* = 3 biological replicates per group.

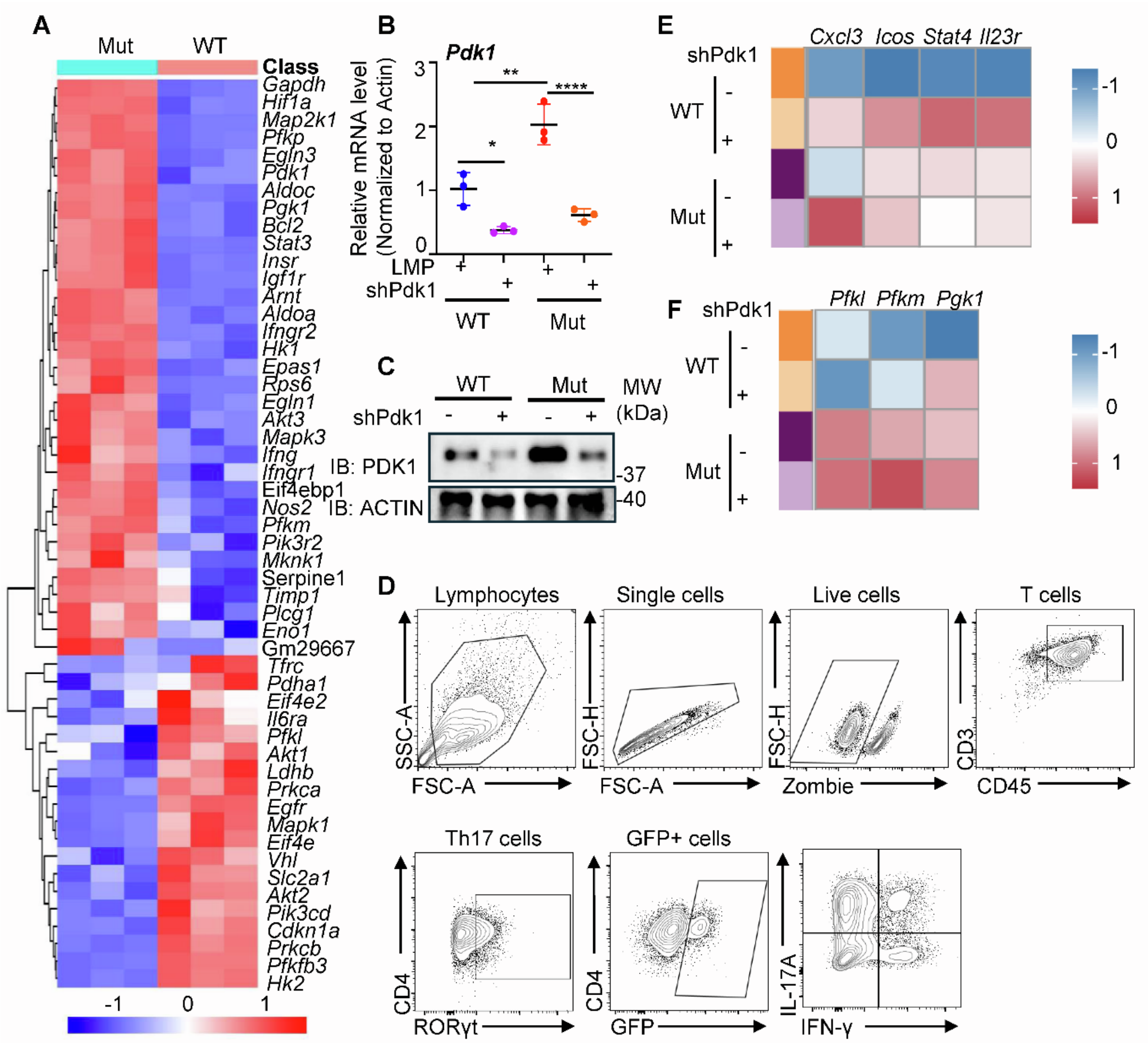

Figure S7. RORγt^{N275D}-HIF-1α elevates PDK1 expression, related to Figure 7.

(A) Heatmap of HIF-1α signaling pathway-associated gene expression from RNA-seq comparing WT and RORγt^{N275D} Th17 cells. **(B)** qRT-PCR validation of *Pdk1* knockdown efficiency in Th17 cells transduced with vector or shRNA retrovirus (normalized to Actin). **(C)** Immunoblot validation of *Pdk1* knockdown efficiency in Th17 cells transduced with vector or *Pdk1* shRNA retrovirus. MW, molecular weight (kDa). **(D)** Gating strategy for flow cytometric analysis of WT or mutant pathogenic Th17 cells transduced with vector or shRNA retrovirus. **(E-F)** Heatmaps showing genes that cannot be rescued by *Pdk1* knockdown in pTH17 cells. WT and Mut pTH17 cells were independently transduced with Pdk1 shRNA–expressing retrovirus (or control), and the expression of pathogenic signature genes (E) and metabolism-associated enzyme genes (F) is shown. Statistical significance was determined by one-way ANOVA. *, *P* < 0.05; **, *P* < 0.01; ****, *P* < 0.0001; ns, not significant. Data are presented as mean ± SD from *n* = 3 biological replicates per group

Table S1. List of primers used in this study for qRT-PCR or ChIP analysis, related to Star Methods: Quantitative real-time PCR and Chromatin Immunoprecipitation.

Gene name	Forward primer (5' - 3')	Reverse Primer (5' - 3')
Ccl5	GCTGCTTTGCCTACCTCTCC	TCGAGTGACAAACACGACTGC
Ifng	ATGAACGCTACACACTGCATC	CCATCCTTTTGCCAGTTCCTC
Lagls3	AGACAGCTTTTCGCTTAACGA	GGGTAGGCACTAGGAGGAGC
Il3	GGGATACCCACCGTTTAACCA	AGGTTTACTCTCCGAAAGCTCTT
Stat4	TGGCAACAATTCTGCTTCAAAAC	GAGGTCCCTGGATAGGCATGT
Lag3	CTGGGACTGCTTTGGGAAG	GGTTGATGTTGCCAGATAACCC
Gzmb	CCACTCTCGACCCTACATGG	GGCCCCCAAAGTGACATTTATT
Il9	ATGTTGGTGACATACATCCTTGC	TGACGGTGGATCATCCTTCAG
Il22	ATGAGTTTTTCCCTTATGGGGAC	GCTGGAAGTTGGACACCTCAA
Cxcl3	GAGACCATCCAGAGCTTGACG	CCTTGGGGGTTGAGGCAAACTT
Il23r	TTCAGATGGGCATGAATGTTTCT	CCAAATCCGAGCTGTTGTTCTAT
Ifngr1	CTGGCAGGATGATTCTGCTGG	GCATACGACAGGGTTCAAGTTAT
Hif1a	GTCCCAGCTACGAAGTTACAGC	CAGTGCAGGATACACAAGGTTT
Pdk1	GGACTTCGGGTCAGTGAATGC	TCCTGAGAAGATTGTCGGGGA
Eno1	TGCGTCCACTGGCATCTAC	CAGAGCAGGCGCAATAGTTTTA
Gapdh	TCCTGAGAAGATTGTCGGGGA	TCCTGAGAAGATTGTCGGGGA
Hk2	TGATCGCCTGCTTATTCACGG	AACCGCCTAGAAATCTCCAGA
Aldoa	TGGCGAGACTACTACCCAAGG	GGGGCGAGGGAGTATGTTTC
Aldoc	AGAAGGAGTTGTCGGATATTGCT	TTCTCCACCCCAATTTGGCTC
Pgk1	ATGTCGCTTTCCAACAAGCTG	GCTCCATTGTCCAAGCAGAAT
Pfkm	TGTGGTCCGAGTTGGTATCTT	GCACTTCCAATCACTGTGCC
Pfkl	GGAGGCGAGAACATCAAGCC	CGGCCTTCCCTCGTAGTGA
Pfkfb3	CCCAGAGCCGGGTACAGAA	GGGGAGTTGGTCAGCTTCG
Ldhb	CATTGCGTCCGTTGCAGATG	GGAGGAACAAGCTCCCGTG
Pdha1	GAAATGTGACCTTCATCGGCT	TGATCCGCCTTTAGCTCCATC
Ifngr1 promoter	TGATCCGCCTTTAGCTCCATC	TCCTGCGCGAGGGACACCTC
Pdk1 promoter	CGCGTTTGGATTCCGTG	CCAGTTATAATCTGCCTTCCCTATTATC
Hbb promoter	GCTCTGGGTACTCCCTCTGA	GCAAATGTGTTGCCAAAAAG
